# Factors Influencing Biofilm Formation of *Salmonella* spp. and the Biofilm-Degrading Potential of Essential Oils

**DOI:** 10.3390/foods15091574

**Published:** 2026-05-03

**Authors:** Anita Seres-Steinbach, Péter Szabó, Viktória Balázs Lilla, Eszter Riethmüller, Ama Szmolka, Krisztián Bányai, György Schneider

**Affiliations:** 1 Department of Medical Microbiology, Medical School, University of Pécs, H-7624 Pécs, Hungary; seres-steinbach.anita@edu.pte.hu; 2 Department of Geology and Meteorology, Faculty of Sciences, University of Pécs, Ifjúság Str. 6, H-7624 Pécs, Hungary; sz.piiit01@gmail.com; 3 Environmental Analytical and Geoanalytical Research Group, Szentágothai Research Center, Ifjúság Str. 20., H-7624 Pécs, Hungary; 4 Department of Pharmacognosy, Faculty of Pharmacy, University of Pécs, H-7624 Pécs, Hungary; balazs.viktoria@gytk.pte.hu; 5 Department of Pharmacognosy, Faculty of Pharmacy, Semmelweiss University, H-1085 Budapest, Hungary; riethmuller.eszter@semmelweis.hu; 6 HUN-REN Veterinary Medical Research Institute, Hungária Krt. 21, H-1143 Budapest, Hungary; szmolka.annamaria@vmri.hun-ren.hu; 7 Department of Medical Biology, Medical School, University of Pécs, H-7624 Pécs, Hungary; banyai.krisztian@univet.hu; 8 Department of Pharmacology and Toxicology, University of Veterinary Medicine, H-1078 Budapest, Hungary

**Keywords:** *Salmonellae*, biofilm formation, essential oil, biofilm inhibition, biofilm degradation

## Abstract

The formation of biofilms by Salmonella is of considerable interest to the food production and medical industries. This study investigated the effects of a carrier medium (Luria–Bertani, Mueller–Hinton II, Brain Heart Infusion or chicken meat juice), temperature (14 °C, 23 °C or 37 °C) and surface type (adhesive, non-adhesive or suspension plate) on biofilm formation in 16 different Salmonella isolates belonging to the serovars *S.* Enteritidis (five isolates), *S.* Infantis (five isolates) and *S.* Typhimurium (six isolates). Chicken meat juice was found to have a moderate yet balanced supportive effect, while Mueller–Hinton II (MH-II) medium drastically supported biofilm formation at low temperatures, albeit with significant variation among the isolates. Temperature and medium also affected the antibacterial, biofilm inhibitory and destructive effects of essential oils. At 14 °C and 23 °C, 35% of essential oils exhibited antibacterial activity against Salmonella serovars at a concentration of 0.1%, as determined by the drop plate method. Ajowan, thyme, orange, clove and oregano EOs completely inhibited biofilm formation at a concentration of 0.1%. More than half of the 60 essential oils tested reduced the optical density of mature biofilms (OD: 0.15–0.36) to below 0.05; ajowan, lime, palmarosa, thyme, oregano and clove were the most effective, exhibiting antibacterial, biofilm inhibitory and biofilm destructive effects on all of the investigated Salmonella isolates.

## 1. Introduction

*Salmonella* spp. are among the most common foodborne pathogens being responsible for 1.3 billion cases of gastroenteritis worldwide. Of the seven subspecies, (I, II, IIIA, IIIB, IV, V, and VI) of the two species (*S. bongori* and *S. enterica*), six (I, II, IIIA, IIIB, IV, and VI) have the capacity to attack warm-blooded animals. Based on the flagellar, lipopolysaccharide (LPS) and capsule-like matrix antigens, more than 2600 serovariants can be distinguished in *S. enterica,* which is associated with salmonellosis in humans. This infection causes symptoms such as abdominal pain, headache, and diarrhea, affecting approximately 9.8 million people worldwide, 155,000 of whom die. The most frequent serovars responsible for food-related zoonotic infections in humans are *S.* Enteritidis, *S.* Typhimurium and *S.* Infantis [1].

One important risk factor for human infection is the consumption of chicken and turkey [2,3]. During exposure, Salmonella enters the digestive system and, due to its tolerance of acidic environments, survives in the stomach. It then enters the intestine and adheres to intestinal epithelial cells in the small intestine [4]. Here, the pathogen uses its type III secretion systems, encoded on the Salmonella pathogenicity islands 1 and 2 (SPI-1 and SPI-2), to inject effectors into the eukaryotic cells, thereby modifying their internal structure and function to facilitate bacterial entry, intracellular survival, and proliferation [5,6]. Additional factors contributing to this process are encoded on islands such as SPI-3, SPI-4, SPI-5 and SPI-6 [1,7,8,9].

Treatment with antibiotics is becoming an increasing challenge, as the uncontrolled use of antibiotics in livestock farming over the last few decades has led to the emergence of increasingly resistant strains of *Salmonellae* [10]. Certain isolates can hydrolyse β-lactam antibiotics, such as penicillin and cephalosporin. They can also pump out macrolides, β-lactams, fluoroquinolones, oxazolidinones, fourth-generation cephalosporins, and, most recently, carbapenems, using their efflux pumps [11,12]. Nevertheless, a significant proportion of the isolates have the ability to form biofilms [13,14,15], a feature confirmed to play a crucial role in survival on both biotic [16,17,18] and abiotic surfaces [19,20,21,22]. For example, biofilm formation in *Salmonella* Typhimurium increases its survival on meat surfaces [23]. Biofilms can protect bacteria not only from certain antibiotics, but also from disinfectants [24,25].

To address the aforementioned challenge, the importance of prevention should be emphasized as a primary strategy in animal husbandry, slaughterhouses and the production of fresh meat products. Today, oxidizing agents, such as peroxyacetic acid, are widely applied as disinfectants. In addition the potential application of bacteriophages [26], vaccines [27], or probiotics, the use of essential oils is also considered due to their antibacterial properties [28]. One advantage of essential oils over antibiotics is their diverse chemical composition, which gives them a wide range of effects, such as general membrane disruption or more specific inhibitory features, such as inhibition of nucleic acid or protein biosynthesis or blocking enzymatic effects [29]. Despite their potential and practical relevance, the number of studies investigating the sensitivity of Salmonella serovars to essential oils (EOs) is still low [30,31,32,33]. The efficacies of orange [30,34], thyme [31,35], oregano [32] and curcuma [33] against *Salmonella* Enteritidis, *S.* Typhimurium, and *S. Iinfantis* have recently been revealed.

The aim of this study was to expand our knowledge of essential oils with anti-Salmonella properties. For this, we screened the anti-Salmonella activities of 60 essential oils against 16 *Salmonella* isolates belonging to different serovars (*S.* Eenteritidis (5), *S.* Infantis (5) and *S.* Typhimurium (6)). We also investigated EOs that could have practical relevance, focusing not only on their anti-Salmonella features, but also on their ability to inhibit or disrupt biofilms, analyzing their potential as disinfectants. To reveal the major influential factors, the experiments were performed in different media such as Luria–Bertani (LB) broth, Mueller–Hinton II (MH-II) broth, Brain Heart Infusion (BHI) and partially in chicken meat juice (CMJ). For the comparative experiments, different surface qualities were used, such as polystyrene surfaces with different adhesion features and stainless steel, all at different temperatures.

## 2. Materials and Methods

### 2.1. Bacterial Strains, Media

Sixteen different isolates of previously characterized *Salmonella* serovars, such as *S.* Enteritidis, *S.* Infantis and *S.* Typhimurium, were used in this study [36]. Fifteen of these isolates were obtained from chickens in Hungary, while the *S. enterica* subsp. enterica serovar Typhimurium strain 14,028 was a reference strain registered in our collection as 10,383 (Table 1). The isolates were routinely grown on Luria–Bertani (LB) agar plates. Prior to the experiments, individual clones were inoculated into 5 mL of LB broth and incubated for 18 h at 37 °C in a shaking incubator at 120 × rpm. Bacterial cell densities were synchronized to an OD_620 nm_ = 0.2 (approximately 10^8^ CFU/mL) using a spectrophotometer. The resulting bacterial suspensions were used for the downstream processes described below. Mueller–Hinton II (MH-II, Biolab Co., Budapest, Hungary) and Brain Heart Infusion (BHI, Biolab Co., Hungary) liquid media were used for biofilm formation experiments. Chicken meat juice (CMJ) was used for some experiments. CMJ was prepared from fresh chicken breast before the experiments. To 100 g of chicken meat, 200 mL of 0.9% NaCl solution was added and minced. After centrifugation (10,000× *g* for 10 min), the supernatant was sterile filtered, and the resulting meat juice was used for further experiments [37].

### 2.2. Essential Oils

Sixty essential oils were used in this study (Supplementary Table S1), most of which had been previously tested on *Listeria monocytogenes* isolates [37]. The EOs were used in either a concentrated or a diluted form, as indicated in the relevant sections. One and five percent emulsions were prepared from the concentrated essential oils in a 1% Tween 20 solution. Sterility tests were regularly performed on the EO suspensions during the experiments. Quality essential oils were purchased from A.G. Industries (Noida, UP, India). Within the framework of the study, compound compositions were analyzed and their quality was confirmed for 6 EOs: ajowan, clove, lime, palmarosa, oregano and thyme.

### 2.3. Antibacterial Testing of Essential Oils—Drop Plate Method

The antibacterial activity of the 60 essential oils was determined using the drop plate method. For this, the bacterial suspensions were synchronized to an OD_620 nm_ = 0.2. One hundred microliters of the bacterial suspensions was evenly spread onto Columbia Blood Agar base plates using a sterile glass spreader to form bacterial lawns. Five microliters of the 1% and 5% EOs suspensions were then dropped onto the bacterial lawns. The plates were then incubated at 14 °C, 23 °C, and 37 °C and the inhibition zones were measured after 24 h for the samples kept at 23 °C and 37 °C, and after 72 h for the samples incubated at 14 °C. Drop plate experiments were carried out twice on two different days.

### 2.4. Biofilm Formation Tests on Polystyrene and Stainless-Steel Surfaces

The ability of 16 *Salmonella* isolates (Table 1) to form biofilms was assessed at temperatures of 4 °C, 14 °C, 23 °C, and 37 °C. This was done using 96-well polystyrene tissue culture plates (Sarstedt, Nümbrecht, Germany) featuring three surface types with different adhesive properties. These were adhesive (code: 83.3924), complex adhesion (code: 83.3924.300), and suspension cell surfaces (code: 83.3924.500), which are hereafter referred to as A, CAC, and SC, respectively. In addition, stainless-steel mesh (SSM) samples (dimensions: 0.27 × 0.27 × 0.16 mm; area: 25 mm^2^; Metmark Kft, Szekszárd, Hungary) were also used for biofilm testing.

Experiments were conducted in four types of media: LB, MH-II, BHI, and chicken meat juice (CMJ). Essential oils were applied at concentrations of 0.1% and 0.5%, using 1% Tween 20 to increase solubilisation. For the assays, overnight bacterial cultures (adjusted to an OD_620_ = 0.2) were diluted 1:100 in LB, MH-II, or BHI media. From each dilution, 200 µL was dispensed into the wells of 96-well plates. The plates were then incubated at 23 °C or 37 °C for 18 h, at 14 °C for two days or at 4 °C for six weeks.

After incubation, the non-adherent bacteria were gently removed, and the wells were rinsed three times with PBS. The adherent (sessile) cells were fixed using a 2% formalin solution (200 µL; prepared by mixing 28.6 mL of 35% formalin with 471.4 mL PBS) for two minutes. After discarding the fixative, the plates were dried at 37 °C for two hours. The plates were stained with a 0.13% crystal violet solution (consisting of 14.29 mL 35% formalin, 234.41 mL PBS, 1.302 mL 96% ethanol, and 0.325 g crystal violet) for 20 min, followed by three washes with PBS. The bounded dye was then solubilised with 200 µL of a 1% SDS solution (a 1:1 mixture of 50% ethanol and PBS) for two hours. Absorbance was measured at 630 nm using a multimode microplate reader (AMR-100T, Hangzhou, China).

To test for biofilm formation on stainless-steel surfaces, small pieces (1 × 1 cm) were soaked in 95% ethanol for one day and then autoclaved. Each SSS piece was then placed in an individual well of a 24-well polystyrene plate, after which the ability of the bacterial isolates cultured to an OD_620_ of 0.2 and diluted at 1:100 to form biofilms was tested at 4 °C, 14 °C, 23 °C and 37 °C in LB, MH-II and BHI broths. The plates were then incubated for one day at 23 °C and 37 °C, two days at 14 °C and six weeks at 4 °C. After the incubation period, the stainless-steel pieces were transferred to a new sterile 24-well plate, washed three times with PBS and fixed with 2.5% glutaraldehyde for two hours. The fixed bacteria were dehydrated using a graded ethanol series of 50%, 80% and 96%, with each step applied for 30 min [37,38], after which bacterial adhesions and biofilms were visualized using scanning electron microscopy.

Biofilm formation tests were performed in three parallel tests on the same day (one plate and three parallels).

### 2.5. Scanning Electron Microscope (SEM) Analysis

Scanning electron microscopy was used to observe the adhesion and biofilm development of the *Salmonella* Typhimurium isolate 1268 on stainless-steel surfaces (SSSs) and the disruptive potential of ajowan, clove, lime oil (according to BP), palmarosa, oregano and thyme essential oils on mature *S.* Typhimurium biofilms.

One millilitre of an MH-II broth culture of isolate 1268 with an OD_620_ of 0.2 was centrifuged at maximum speed for one minute. The supernatant was then removed, and the pellet was resuspended in 100 µL of undiluted essential oil. The samples were then incubated in the medium together for one hour and 24 h, respectively, before being fixed with 2.5% glutaraldehyde for two hours. The bacterial cells were gradually dehydrated using three applications of ethanol at 50%, 80%, and 96% for 10 min each. The samples were then dried using 50% and 100% hexamethyldisilazane (HMDS) (Sigma-Aldrich, St. Louis, MO, USA) for 30 min. Drying with hexamethyldisilazane was already performed on microscope slides.

Finally, the bacterial cells were mounted on aluminum stubs. Fixed samples on the SSS and microscope slides were coated with a thin layer of gold using a JEOL JFC-1300 auto fine coater (JEOL, Tokyo, Japan). Imaging was conducted using a JEOL JSM-IT500HR SEM (JEOL, Tokyo, Japan) operated in secondary electron detection mode. Micrographs were captured at various magnifications with an accelerating voltage of 5 kV and a probe current of 45 kV [39].

The SEM provided us with qualitative information on the state and coverage of the biofilm biomass. We did not derive quantitative information about the biofilm from the SEM images. The basis for quantification was provided by the OD values obtained from the crystal violet assay performed on the polystyrene surface.

SEM analyses were performed twice after samples were either treated with EO or not (control). The most typical images were presented.

### 2.6. Testing the Biofilm Inhibitory and Desruptory Effects of EOs

Experiments involving biofilm inhibition assays were carried out using the *S.* Typhimurium isolate 1268, always in two parallel complex adhesion cell plates (see below), as described previously (see Section 2.4), with some modifications. In this case, however, essential oils were added to the wells at the start of the incubations at a final concentration of 0.1%. With the previously described biofilm assay, the tests were performed at 23 °C and 37 °C for 24 h, and at 14 °C for 48 h. After incubation, viable cell counts were determined by serial dilutions from one plate, while inhibition of biofilm matrix formation was revealed by applying the crystal violet staining procedure described above to the other plate.

To evaluate the effect of the essential oils on mature biofilms, they were first established in 96-well plates, as described previously (see Section 2.4). After biofilm formation (1 day at 23 °C and 37 °C, and 2 days at 14 °C), the medium and non-sessile cells were gently removed, and wells were rinsed three times with PBS. Then, 200 µL of sterile MH-II/BHI/CMJ fresh medium containing essential oils at a concentration of 0.1% was added to each well. Following 24 h exposure periods at the relevant temperatures, the biofilms were fixed and stained as previously described, and the retained crystal violet was quantified spectrophotometrically.

Additionally, to assess the bactericidal activity post-treatment, samples were taken from wells containing essential oils in MH-II/BHI/CMJ. Colony-forming units (CFUs) were determined to allow for an evaluation and comparison of the antimicrobial efficacy at all three tested temperatures.

Biofilm inhibition and degradation experiments were performed in three parallel tests on the same day (one plate three parallels).

### 2.7. Statistical Analysis

Statistical analyses were performed using JASP version 0.18.3.0. The relationship between temperature and optical density was visualized using marginal effects plots. ANOVA was used to conduct comparisons between temperature, media, isolates, essential oils, essential oil concentrations and surfaces. The most commonly applied significance level in our analyses was *p* < 0.05.

## 3. Results

### 3.1. Antimicrobial Testing—Drop Plate Method

**Figure 1:**
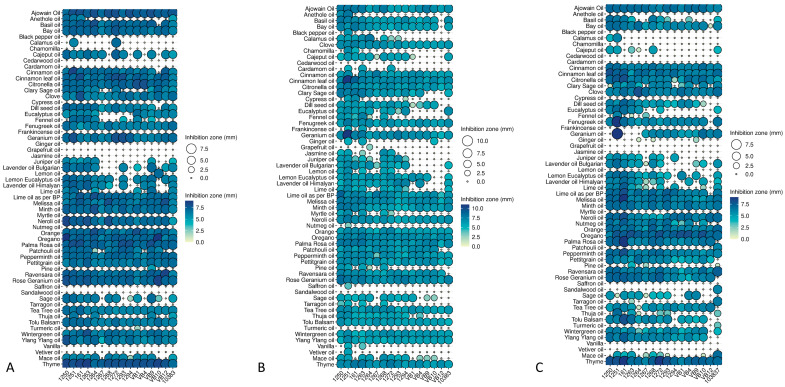

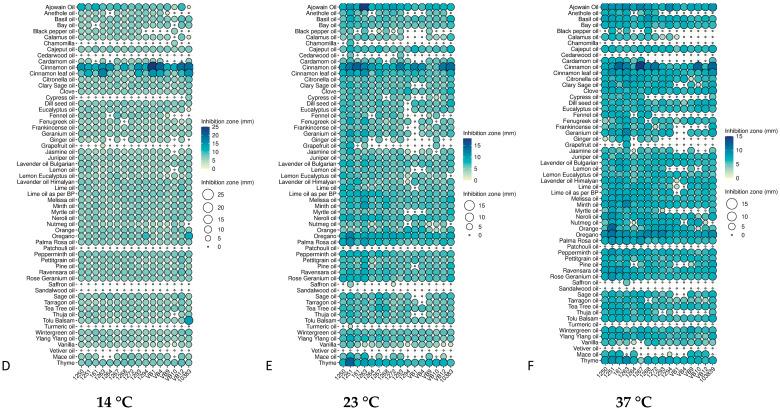
Antibacterial activities of the 60 tested essential oils revealed by the drop plate method at 14 (**A**,**D**), 23 (**B**,**E**) and 37 (**C**,**F**) °C. Bacterial lawns were exposed to 1% (**A**–**C**) and 5% (**D**–**F**) concentrations of essential oils and incubated overnight.

The antibacterial efficacy of 1% essential oil suspensions was tested on the lawns of the 13 *Salmonella* isolates at 14 °C (Figure 1A,D), 23 °C (Figure 1B,E) and 37 °C (Figure 1C,F). The results are presented by using dot plots.

At 14 °C, 35% (22/60) of the EOs were effective at a 1% concentration, typically showing inhibition zone diameters between 5 and 8 mm. Using 5% concentrations increased the number of effective essential oils, with 63% (38/60) showing a characteristic inhibition zone (typically 6–25 mm) on all *Salmonella* serovariant isolates. Cinnamon proved to be the most effective at this increased concentration, with an inhibition zone of 20–25 mm.

The least effective essential oils at a 1% concentration at 14 °C were black pepper, ginger, grapefruit, jasmine, myrtle, nutmeg, saffron, sandalwood, vanilla, and vetiver. At 5% concentration, the least effective essential oils were cedarwood, grapefruit, patchouli, saffron, sandalwood, and vetiver (Figure 1). The solvent did not have any anti-Salmonella effects.

At 23 °C, 35% (21/60) of the essential oils were effective against all isolates when tested at a concentration of 1%, showing inhibition zone diameters between 4 and 7.5 mm. This value increased to 45% (27/60) when the 5% EO samples were applied. Inhibition zones were typically between 6 and 15 mm.

The inhibition patterns of the zone diameters were very similar to the results of the tests performed at 14 °C, which showed that cinnamon was the most effective, with inhibition zone diameters of 6–11 mm at 1% and 6–11 mm at 5%. However, this was 10 mm less than the results obtained at the lower temperature. Oregano and thyme were also effective at concentrations of 1% (O: 6–8 mm; T: 6–8 mm) and 5% (o: 7–12 mm; T: 7–15 mm).

At a concentration of 1%, the least effective essential oils were cedarwood and sandalwood, which did not produce measurable inhibition zones against any of the tested isolates. At a concentration of 5%, however, cedarwood produced a 6 mm inhibition zone against isolates 1251 and 1293. Black pepper at a concentration of 1% was only effective against isolate 1251; at a concentration of 5%, however, it produced a measurable zone of inhibition against seven other isolates. This increase in efficacy was observed not only with black pepper, but also with 21 other essential oils, including anethole (from 0–6 mm to 0–8 mm), basil (from 0–8 mm to 6–9 mm), cajeput (from 0–6.5 mm to 6–8 mm) grapefruit (from 0–3 mm to 0–8 mm), juniper (from 0–7.2 mm to 5–7 mm), lavender (from 0–7.5 mm to 6–8 mm), nutmeg (from 0–3 mm to 0–7 mm) and vanilla (from 0–5 mm to 2–7 mm) (Figure 1).

At 37 °C, 21% (13/60) of the 1% essential oil samples were effective against the tested isolates, compared to 58% (29/60) with the 5% concentration samples. In terms of inhibition zone, the values doubled. With 1% concentrations (typically between 5 and 7.5 mm), it remained to difficult to select the most effective essential oil, but with 5% concentrations, cinnamon was the most effective, with an inhibition zone of up to 15 mm. The least effective essential oils at a 1% concentration were black pepper, cedarwood, cardamom, cypress, grapefruit, jasmine, lemon, myrtle, saffron, sandalwood, vanilla, and vetiver. In the case of a 5% concentration, the essential oils typically produced an inhibition zone of between 6 and 15 mm. However, chamomilla, cedarwood, patchouli, sandalwood, and vetiver did not show any inhibitory effect on any of the tested isolates (Figure 1).

Interestingly, patchouli essential oil was more effective at the lower concentration than at 5%.

### 3.2. Biofilm Formation Ability Tests on Polystyrene Surfaces—Statistical Evaluation

**Figure 2:**
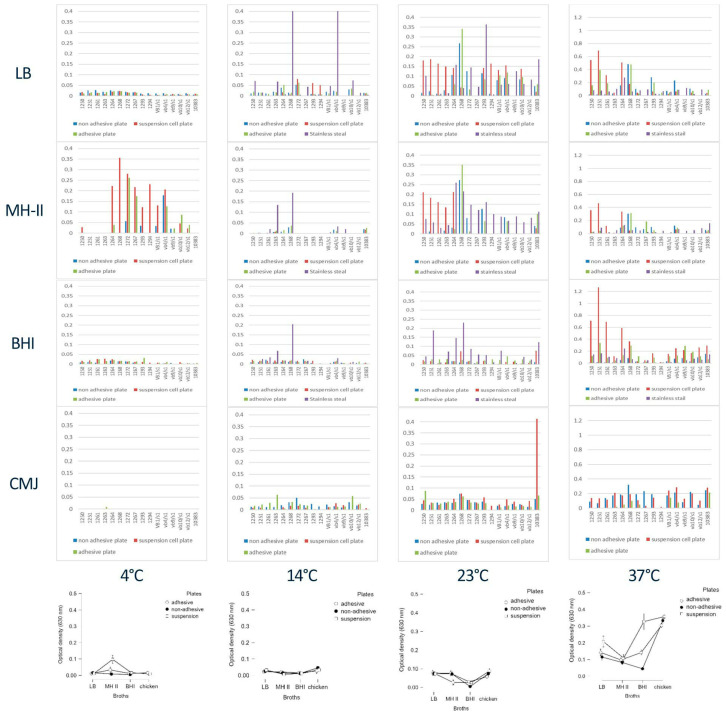
Biofilm formation of the 16 investigated Salmonella isolates at 14, 23 and 37 °C, in LB, MH-II, BHI, and CMJ on different adhesive surfaces and evaluation the biofilm formation results with statistical analyses (bottom line). (Data are presented in Supplementary Table S2).

The biofilm formation abilities of the 16 investigated *Salmonella* isolates were examined in various combinations of temperatures (4 °C, 14 °C, 23 °C and 37 °C), carrier media, such as Luria–Bertani (LB), Brain Heart Infusion (BHI), Mueller–Hinton II (MH-II) and chicken meat juice (CMJ), and surfaces (adhesive, complex-adhesive, and suspension polystyrene, as well as stainless-steel surfaces). The most striking effect on biofilm formation was observed at 4 °C, where the formation of biofilms by several isolates, typically belonging to *S.* Typhimurium and *S.* Infantis, was significantly enhanced, albeit only in the presence of MH-II (Figure 2).

Regarding to the isolates, representatives of *S.* Enteritidis showed characteristically larger matrix formations at 23 °C, but only in LB and MH-II medium when the suspension plate was used. Furthermore, this feature was also evident at 37 °C. Isolate 1263 was the least active. The most consistent matrix formation was observed in CMJ when applied at 14 °C and 23 °C, and biofilm formation was much more pronounced at 23 °C, but showed strong medium dependence, primarily being positively influenced by LB and MH-II media.

At 37 °C, the ability of the isolates to form biofilms in LB and MH-II media decreased drastically, while, in contrast, this feature improved in some isolates if BHI was the carrier medium. Biofilm formation by the 16 Salmonella isolates appeared more consistent and intense at 23 °C and 37 °C (Figure 2).

Altogether, 576 data points were obtained from the biofilm formation experiments carried out at 4 °C, 14 °C, 23 °C, and 37 °C, for which the optical density values were as follows: 0.019 ± 0.074 at 4 °C, 0.022 ± 0.048 at 14 °C, 0.056 ± 0.068 at 23 °C, and 0.188 ± 0.182 at 37 °C. In order to examine what influenced biofilm formation and whether the differences were significant between the various factors, Levene’s test within ANOVA was performed, which yielded a *p*-value of <0.001 (F:7.844), indicating that the assumption of homoscedasticity was violated and the variances across groups were unequal. For the Shapiro–Wilk test *p* < 0.001, so we used the Kruskal–Wallis test (Table 2). Based on the Kruskal–Wallis test, plates (df:2), strains (df:15), broths (df:3) and temperatures (df:3) all had *p* < 0.001. Based on Dunn’s post hoc test, there was a significant difference between adhesive and non-adhesive plates (*p* < 0.001; z:3.918; pbonf and pholm: *p* < 0.001), and there was a significant difference between adhesive and suspension plates (*p* = 0.034; z = 2.116); however, considering the pbonf (*p* = 0.103) and pholm (*p* = 0.069) corrections, there was no significant difference, and there was also no significant difference between the non-adhesive and suspension plates (*p* = 0.071; z = −1.8).

Based on Dunn’s post hoc analysis, several significant differences were observed among the different media (LB, MH II, BHI, and chicken).

There was no significant difference between the LB and MH II culture media (z = 1.829, *p* = 0.067). This suggests that there is no difference between these two culture media; they influence biofilm formation to the same extent. In contrast, a significant difference is observed between LB broth and BHI (z = 5.071, *p* < 0.001). Similarly, there is a significant difference between LB broth and CMJ (z = −6.661, *p* < 0.001). There is also a significant difference between MH II and BHI (z = 3.243, *p* = 0.001). Significant differences are also observed between MH II and CMJ (z = −8.489, *p* < 0.001) and between BHI and CMJ (z = −11.732, *p* < 0.001).

When examining the differences between temperatures, significant differences were revealed across the board. Comparing biofilm formation at 4 °C with the other temperature groups yields the following values: 4 vs. 14 °C: z = −4.983; 4 vs. 23 °C: z = −13.845; and 4 vs. 37 °C: z = −25.527; *p* < 0.001 in all cases. The 14 °C group also showed significant differences compared to the higher-temperature groups (14 vs. 23 °C: z = −8.862; 14 vs. 37 °C: z = −20.544; both *p* < 0.001). There was also a significant difference between the 23 °C and 37 °C groups (z = −11.681; *p* < 0.001).

**Table 2:** Stepanović biofilm classification.

Broth	Temperature	ODc	No Biofilm	Weak	Moderate	Strong
LB	4 °C	0.0779	OD ≤ 0.0779	0.0779 < OD ≤ 0.1558	0.1558 < OD ≤ 0.3119	OD > 0.3119
LB	14 °C	0.1052	OD ≤ 0.1052	0.1052 < OD ≤ 0.2104	0.2104 < OD ≤ 0.4205	OD > 0.4205
LB	23 °C	0.1146	OD ≤ 0.1146	0.1146 < OD ≤ 0.2293	0.2293 < OD ≤ 0.4586	OD > 0.4586
LB	37 °C	0.0840	OD ≤ 0.0840	0.0840 < OD ≤ 0.1680	0.1680 < OD ≤ 0.3360	OD > 0.3360
BHI	4 °C	0.0800	OD ≤ 0.0800	0.0800 < OD ≤ 0.1600	0.1600 < OD ≤ 0.3200	OD > 0.3200
BHI	14 °C	0.0842	OD ≤ 0.0842	0.0842 < OD ≤ 0.1684	0.1684 < OD ≤ 0.3364	OD > 0.3364
BHI	23 °C	0.0916	OD ≤ 0.0916	0.0916 < OD ≤ 0.1832	0.1832 < OD ≤ 0.3669	OD > 0.3669
BHI	37 °C	0.0885	OD ≤ 0.0885	0.0885 < OD ≤ 0.1771	0.1771 < OD ≤ 0.3542	OD > 0.3542
MH II	4 °C	1.1211	OD ≤ 1.1211	1.1211 < OD ≤ 2.2421	2.2421 < OD ≤ 4.4843	OD > 4.4843
MH II	14 °C	0.0897	OD ≤ 0.0897	0.0897 < OD ≤ 0.1793	0.1793 < OD ≤ 0.3586	OD > 0.3586
MH II	23 °C	0.1433	OD ≤ 0.1433	0.1433 < OD ≤ 0.2866	0.2866 < OD ≤ 0.5733	OD > 0.5733
MH II	37 °C	1.0916	OD ≤ 1.0916	1.0916 < OD ≤ 2.1831	2.1831 < OD ≤ 4.3662	OD > 4.3662
chicken	4 °C	2.4852	OD ≤ 2.4852	2.4852 < OD ≤ 4.9705	4.9705 < OD ≤ 9.9410	OD > 9.9410
chicken	14 °C	0.1129	OD ≤ 0.1129	0.1129 < OD ≤ 0.2257	0.2257 < OD ≤ 0.4514	OD > 0.4514
chicken	23 °C	0.1339	OD ≤ 0.1339	0.1339 < OD ≤ 0.2678	0.2678 < OD ≤ 0.5357	OD > 0.5357
chicken	37 °C	0.5363	OD ≤ 0.5363	0.5363 < OD ≤ 1.0727	1.0727 < OD ≤ 2.1453	OD > 2.1453

**Figure 3:**
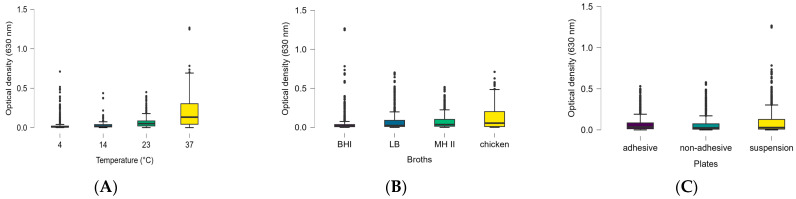
Effect of (**A**) temperature, (**B**) growth media and (**C**) surface qualities on the biofilm formation of the tested Salmonellae isolates, plotted on boxplot diagrams.

Statistical analysis of the three influential factors investigated revealed that, in terms of temperature, 37 °C was optimal; in terms of medium, CMJ was optimal; and, in terms of medium, the suspension plates were optimal for matrix formation (Figure 3A,B). However, marked individual differences could be detected in all cases, which somewhat balances the results, except in the case of temperature and plates, where the CMJ generally performed better at higher temperatures (Figure 3A) than at lower temperatures.

### 3.3. Biofilm Formation Ability on Stainless Steel—SEM Analysis

**Figure 4:**
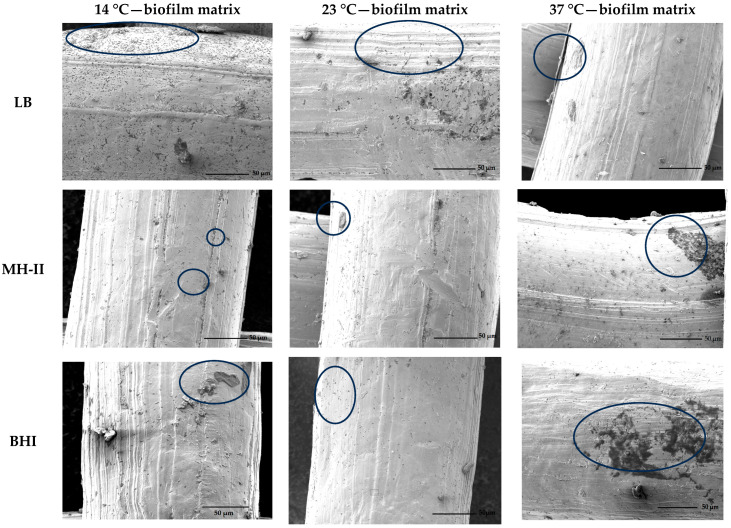
The adhesion and biofilm forming capacity of the *Salmonella* Thyphimurium isolate 1268 on stainless steel mesh visualized by using scanning electron microscope. Diffusely adhered units of bacteria and the loosely organized groups of bacteria are highlighted by circling. Bacterial Sizes of cale bars are 50 µm.

The biofilm-forming ability of the *Salmonella* Typhimurium isolate 1268 on a stainless-steel surface was tested and visualized using scanning electron microscopy. The SEM images showed that this isolate had the potential to adhere to the metal surface at all three of the investigated temperatures (14 °C, 23 °C and 37 °C), regardless of whether LB, BHI or MH-II media were used for the experiment. Regardless of the carrier medium, biofilm formation was the most extensive at 37 °C (Figure 4), although smaller or larger clusters of bacteria were also observed at lower temperatures in the case of BHI and MH-II. At 14 °C, a large number of segregated adherent bacterial cells were visible, and some continuous layers could also be observed (Figure 4).

### 3.4. Biofilm Inhibitory Effects of EOs—Statistical Evaluation

The inhibitory effects of the 60 essential oils on the biofilm of *Salmonella* Typhimurium isolate 1268 were tested at temperatures of 4 °C, 14 °C, 23 °C, and 37 °C, in MH-II and BHI media. The essential oils were tested in non-adhesive 96-well tissue culture plates (code: 83.3924.300) with a final EO concentration of 0.1%. Twenty-four hours were allowed for biofilm formation, during which isolate 1268 was co-incubated with the EOs.

**Figure 5:**
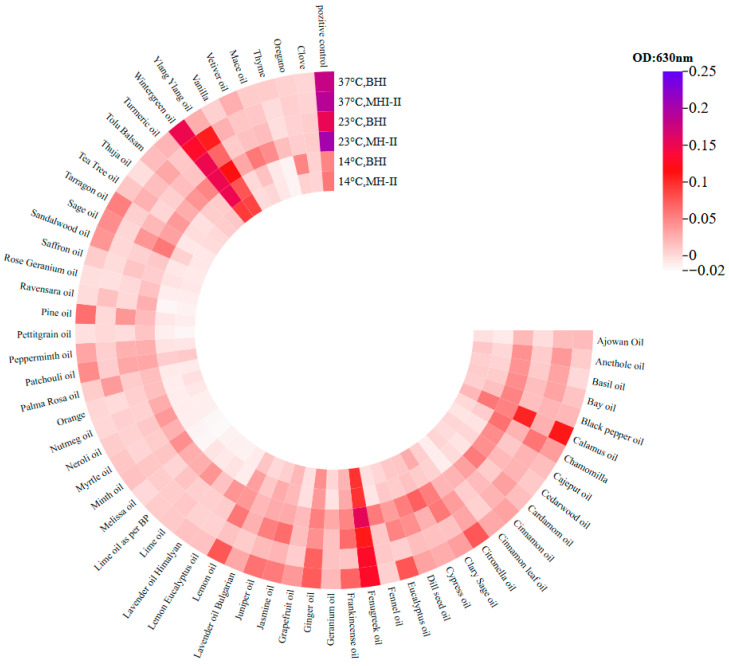
Cumulative diagram of the biofilm inhibitory potential of 60 essential oils on the *Salmonella* Typhimurium isolate 1268. Bacterium cells were grown in MH-II and BHI in the presence of 0.1% EOs, at 14 °C, at 23 °C and at 37 °C for 24 h. The formed biofilm matrices were stained, resolubilized and evaluated by measuring their optical density values, as shown in the heatmap (https://www.chiplot.online/ (accessed on 1 Februray 2024). Optical densities are represented by colours. The darker the colour, the higher the OD value at 630 nm.

Incubation of the bacterial cells with the EOs at 14 °C resulted in the complete inhibition of biofilm formation by 30 out of the 60 oils tested, including ajowan, cajeput, cardamom and cinnamon, regardless of whether the carrier medium was MH-II or BHI (Figure 5). In all these cases, the formed biofilm matrix was less than 50% of the control value (see Supplementary Table S3). For 11 essential oils (basil, bay, citronella, cypress, dill seed, frankincense, ginger, jasmine, patchouli, tolu, turmeric and clove), inhibition was independent of the quality of the presenting medium. Consequently, the results were similar or almost identical in both media, although differences were revealed when comparing them to each other (Figure 5). The third group of essential oils (black pepper, calamus, chamomile, eucalyptus, juniper, lavender, lemon, sage and vetiver) showed that the medium inevitably influenced the outcome, with MH-II being a much stronger inhibitor than BHI. In contrast, BHI did not support the inhibitory effect of calamus or oregano oils at all, whereas MH-II did. Interestingly, the fifth group of oils (fenugreek, wintergreen and ylang ylang) were found to support biofilm formation by the *S.* Typhimurium isolate 1268 compared to the control.

At 23 °C, *S.* Typhimurium isolate 1268 biofilm formation was three times higher than at 14 °C, reaching 0.15 and 0.2 OD in BHI and MH-II, respectively. At this temperature, none of the aforementioned essential oils (fenugreek, wintergreen or ylang ylang) supported biofilm formation. However, at 23 °C, they still exhibited the lowest inhibitory effects on the biofilm formation of the investigated isolate (Figure 5). BHI was found to inhibit biofilm formation more effectively; however, in some cases (calamus, citronella, frankincense, jasmine, juniper, lemon and pine), MH-II exhibited a moderately or distinctly superior inhibitory effect compared to BHI. No influential effects were observed on the inhibitory features of clary sage, patchouli, peppermint, oregano or clove, and no inhibitory effect was observed for wintergreen in either MH-II or BHI.

At 37 °C, the pattern of inhibitory effects was similar to that at 23 °C in that calamus, fenugreek, wintergreen and ylang ylang exhibited the lowest inhibitory effects on *S.* Typhimurium isolate 1268 biofilm formation. Furthermore, the highest biofilm formation rate was observed in the control group, regardless of the carrier medium applied (Figure 5). Overall, the inhibitory effect of MH-II on biofilm formation decreased compared to the values at 23 °C. Temperature significantly affected matrix formation, particularly when MH-II was the carrier medium (Figure 5).

To reveal whether the optical density values of the crystal violet stainings are correlated with the number of living cells, parallel experiments were performed and the number of living cells was determined after incubation.

At 14 °C, the bacterial count in the positive control at the end of the 24th h was 8 log CFU/mL. Co-incubation experiments demonstrated that ajowan, thyme, orange, clove and oregano essential oils completely inhibited biofilm formation at all tested temperatures (14 °C, 23 °C, and 37 °C) in both growth media and at both applied concentrations. These oils also significantly reduced the number of viable cells by 7–8 orders of magnitude, demonstrating an effectiveness of 90–100%. In total, 0.1% essential oils were able to remove live bacteria added to the culture medium, thereby preventing biofilm formation.

The solvent Tween 20 did not have any anti-Salmonella effects in the biofilm inhibition assay using essential oils. We obtained and analyzed 360 data points without a positive control at 14 °C, 23 °C, and 37 °C, for which the optical density values were as follows: 0.02 ± 0.048 at 14 °C, 0.056 ± 0.068 at 23 °C, and 0.188 ± 0.182 at 37 °C. The optical density of the positive control was 0.054 ± 0.005 at 14 °C, 0.178 ± 0.05 at 23 °C, and 0.1845 ± 0.031 at 37 °C.

Based on the significance analysis, the effectiveness of the essential oil was not influenced by the culture medium used (*p* = 0.703), while there was a significant difference between the essential oils used and the temperatures used (*p* < 0.001).

### 3.5. Mature Biofilms Degradation Abilities of the Essential Oils—Statistical Evaluation

**Figure 6:**
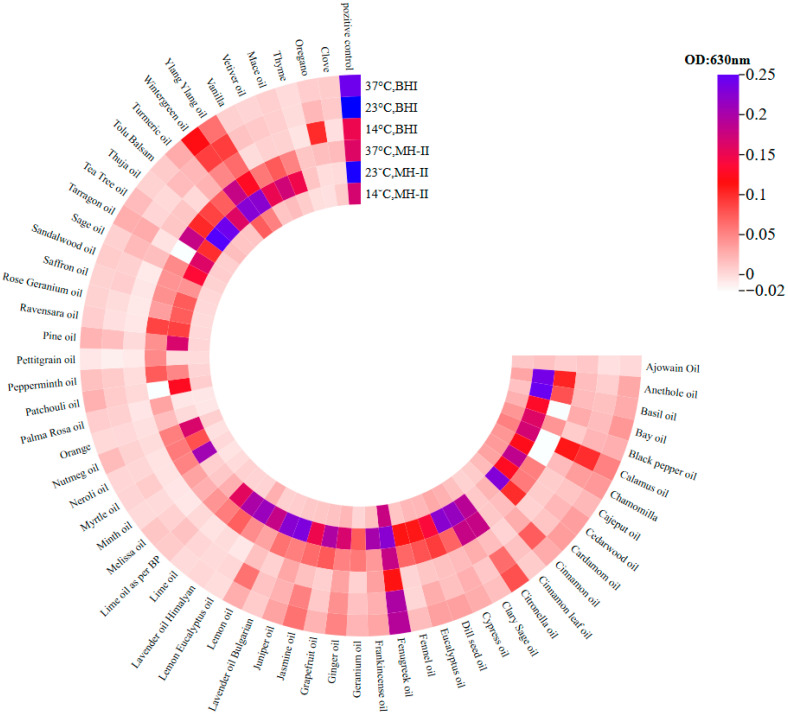
Comparison the biofilm disruption effects of the 0.1% concentrations of the 60 essential oils, investigated on the mature biofilm of *Salmonella* Typhimurium isolate 1268, at 14 °C, 23 °C, and 37 °C in MH-II and BHI media. Darker colours represent fields thicker in the biofilm (https://www.chiplot.online/).

The ability of 60 essential oils to degrade mature biofilms was investigated in MH-II and BHI broth at temperatures of 14 °C, 23 °C and 37 °C, with a final concentration of 0.1%. Based on the results, ajowan, cinnamon, oregano, clove, thyme, and orange essential oils were the most effective, having completely reduced the mature biofilm matrix within 24 h at all three temperatures. In these cases, no viable cells could be detected in the wells of the 96-well complex adhesion cell (CAC) tissue culture plates (Figure 6).

Biofilm-degrading activity was observed in MH-II medium at 14 °C, 23 °C and 37 °C for the following essential oils: ajowan, cinnamon bark and leaf, orange, palmarosa, thyme, oregano, mint, melissa and clove. Compared to the positive control (OD: 0.16–0.62), the optical density values were lower than 0.032, indicating a marked reduction in biofilm formation. Biofilm degradation was most effective at 14 °C and least effective at 23 °C in MH-II.

Biofilm degradation was investigated in BHI broth at 14 °C using various essential oils at a concentration of 0.1%. Based on these results, the most effective essential oils were ajowan, cinnamon, oregano, clove, thyme, petitgrain, and orange, which achieved complete microbial reduction (0 CFU/mL).

The biofilm-degrading activity of the essential oils was markedly more effective in BHI medium. More than half of the 60 EOs tested reduced the optical density of the biofilms (OD: 0.15–0.36) to below 0.05. The most effective essential oils were ajowan, lemon eucalyptus, Himalayan lavender, lime, mint, neroli, orange, palmarosa, petitgrain, ravensara, rose geranium, thyme, oregano, melissa and clove. The effectiveness of the essential oils in removing biofilms was not significantly affected by the applied temperatures.

The Tween20 solvent did not have any anti-Salmonella effect. During biofilm degradation using essential oil, our data without the positive control yielded values of 360–360-360 at each temperature (14 °C, 23 °C, 37 °C), with the following optical density values: 0.014 ± 0.028 at 14 °C, 0.077 ± 0.090 at 23 °C, and 0.042 ± 0.047 at 37 °C.

The optical density of the positive control was 0.159 ± 0.083 at 14 °C, 0.49 ± 0.19 at 23 °C, and 0.20 ± 0.061 at 37 °C.

**Figure 7:**
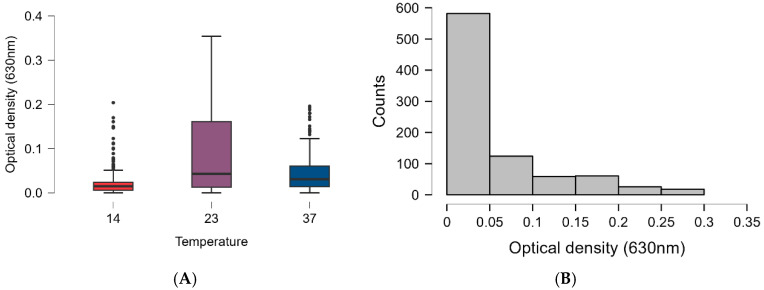
The temperature dependence and distribution of the biofilm degradation ability of the 60 tested essential oils have revealed that (**A**) the process was the most effective at 14 °C and 37 °C, while at 23 °C results diverged, while (**B**) the most frequent values fell between 0.0 and 0.05 OD densities.

Overall, essential oils were most effective at 14 °C, followed by 37 °C, while they were least effective at 23 °C (Figure 7). The optical density values varied the most at 23 °C, where we can see the highest interquartile range (Figure 7A). The distribution graph clearly shows that the most common OD values are between 0.0 and 0.05 OD, which represented almost 55% of all data. The remainder of the data series was distributed between 0.05 and 0.0354 (Figure 7B).

**Figure 8:**
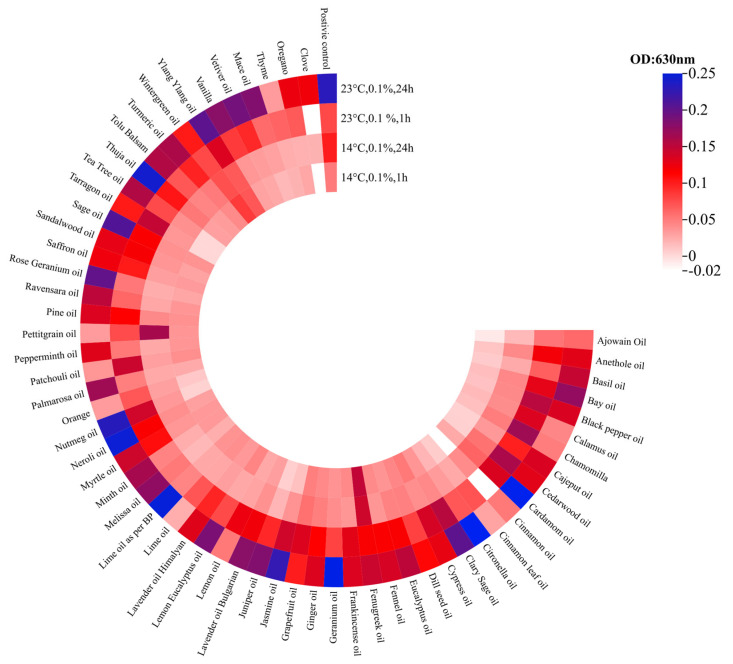
Comparison of the biofilm’s disruption effects of the 0.1% concentrations of the 60 essential oils, investigated on the mature biofilm of the *Salmonella* Typhimurium isolate 1268, at 14 °C and 23 °C in CMJ. Darker colours represent fields thicker in the biofilm (https://www.chiplot.online/).

The degradation of mature biofilms formed in CMJ was investigated at temperatures of 14 °C and 23 °C. The efficacy of these essential oils was lower at 23 °C than at 14 °C. The optical density value of the biofilm was reduced by calamus, chamomilla, and patchouli essential oils, falling to OD_620_ 0.1–0.15 after the first hour and decreasing further to OD_620_ 0.05 after 24 h. No significant difference was observed in the optical density values after 1 or 24 h of incubation with ajowan essential oil (OD_620_ 0.053–0.059). Cinnamon, lime, orange, petitgrain and thyme essential oils reduced the biofilm to an OD of 0.07 within one hour. After a further 23 h of incubation, the measured biofilm decreased to an OD of 0.025–0.033. Of the 60 EOs tested, 43 were less effective after 24 h than after 1 h of incubation. At 14 °C, the essential oils were significantly more effective. Thirty-three of the sixty essential oils were less effective after 24 h of incubation than after one hour. Thirty-four essential oils reduced the biofilm to an OD_620_ < 0.04, including ajowan (OD_620_ < 0.02), juniper (OD_620_ < 0.02), thyme, mint, oregano, palmarosa and clove (OD_620_ < 0.0255). A significantly higher optical density was measured in the presence of other essential oils than in the positive control (fenugreek: OD_620_ > 0.14) (Figure 8).

From the biofilm degradation experiments, we obtained 480 data points from each temperature (14 °C, 23 °C), for which the optical density values were 0.04 ± 0.069 at 14 °C and 0.126 ± 0.123 at 23 °C. When the mean and standard deviation of the optical density values over incubation time were examined, it was found that at 1 h they were in the range of 0.061 ± 0.086, and after 24 h they were 0.105 ± 0.124. After 24 h of incubation, the mean values were higher, which is due to the reduced efficacy of certain essential oils. The standard deviation for the positive control was 0.05 ± 0.007 after 1 h at 14 °C, and 0.1 ± 0.014 after one day of incubation. At 23 °C, the values were 0.078 ± 0.00071 after one hour, and 0.22 ± 0.02 after 24 h.

### 3.6. Biofilm Inhibitory Effect of Essential Oils on Stainless Steel—SEM Analysis

**Figure 9:**
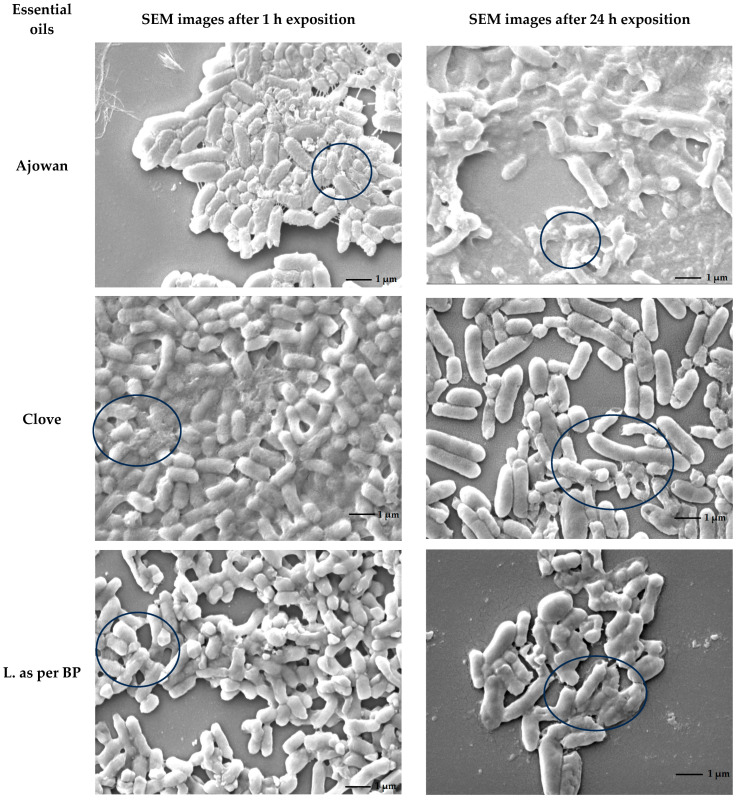

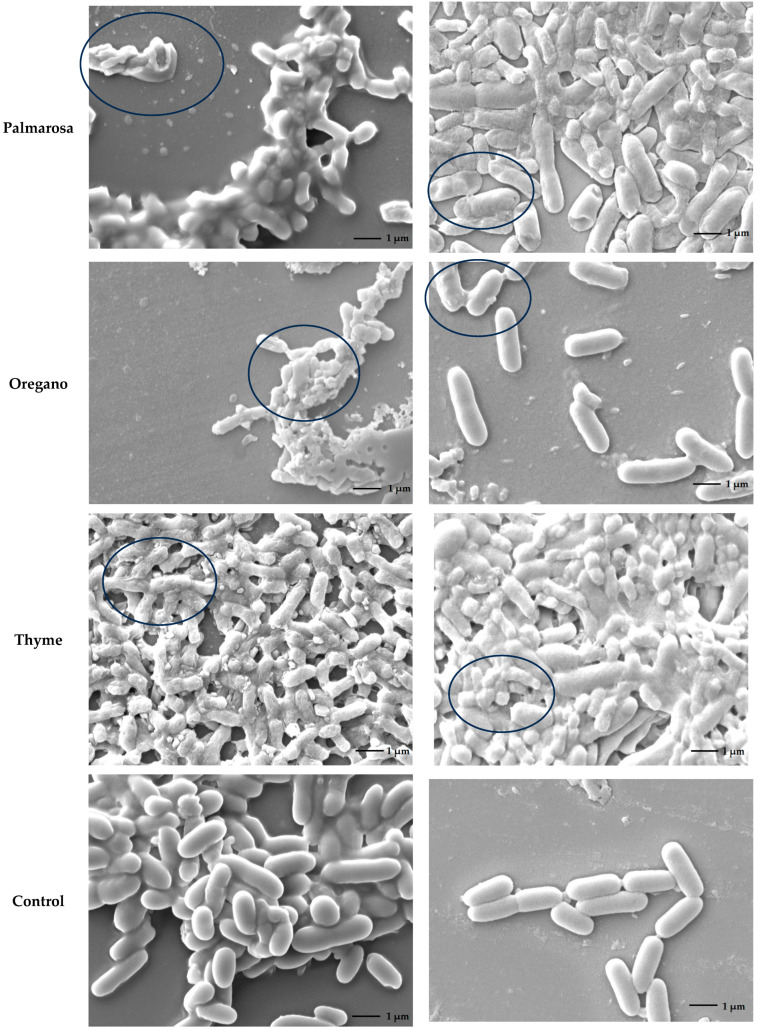
Visualization the morphological changes in *S.* Thyphimurium (isolate 1268) cells after 1 and 24 h incubations in the presence of ajowan, clove, lime, palmarosa, oregano and thyme.

The biofilm inhibitory effects of six essential oils, such as ajowan, clove, lime, palmarosa, oregano and thyme, were revealed on the surface of stainless steel using a scanning electron microscope. Drastic differences could be observed in ajowan-exposed cells after just one hour compared to the control group. These cells exhibited a wrinkled, raisin-like surface and had shrunk in length. After 24 h, the cells had a gelatinous consistency. Both elongated and short, stubby morphotypes were observed. The ajowan-exposed cells appeared dehydrated, whereas the control cells remained intact and rounded with a proper isotonic state (Figure 9).

## 4. Discussion

Despite their characteristic antibacterial properties, a low number of studies have tested the efficacy of essential oils against the members of *Salmonellae*, one of the most important bacterial genera responsible for foodborne diseases [40]. Including isolates from the three most frequent serovariants of the *Salmonella enterica*, namely *S.* Typhimurium, *S.* Infantis and *S.* Enteritidis, in the study was justified by their frequent association with human infections [41,42,43].

Sixty quality-assured essential oils were used in this study. Compositions of the six GC-analyzed EOs have revealed that they show the compositions characteristic for these EOs (Supplementary Figure S1).

For the experiments, the Tween 20 (E432) emulsifier was used, which is approved for the food industry by the European Food Safety Authority and the Food and Drug Administration [44]. Not only is it safe, but it also has good emulsifying properties even at low concentrations. Due to its nonionic nature, it is stable under various pH and ionic strength conditions and exhibits minimal interaction with the components of the food matrix [45]. By using this substance, a homogeneous mixture of essential oils in an aqueous medium could be assured [46].

Practical concentrations of essential oils (EOs) in industrial solutions are generally low, typically ranging from 0.01% to 5% (v/v or w/v) depending on the application method, environment and microorganisms and certainly considering sensory (food) or cost-efficiency limitations [47,48]. Concentrations are often optimized but, because of this information and practical applicability, our experimental setups were designed in this range.

Eliminating adhered cells and thereby reliably preventing biofilm formation or degrading mature Salmonella biofilms, essential oils offer potent alternatives. The lack of comprehensive studies, however, made it reasonable to first screen the anti-Salmonella effects of several EOs, from which 16 distinct Salmonella isolates and 60 EOs were chosen. These oils were recently tested against *Listeria monocytogenes* and, in the framework of the current study, 37 were proven to be effective against the tested Salmonella isolates using the drop plate method (Figure 1) [38]. Although our results showed slight differences in the antibacterial efficacies of the EOs at 14 °C, 23 °C and 37 °C (Figure 1), for example, in the case of lemon, thuja, myrtle and jasmine, the generally balanced antibacterial effects were far less spectacular than those recently observed for another foodborne pathogen, *Listeria monocytogenes*, where bacterial cells were most affected at high (37 °C) and low (4 °C) temperatures [38]. The chemical features of the essential oils (EOs), physiological sensitivities of the target bacterial cells, temperature dependent membrane fluidity, increased volatilised state and proton ionophore ability of EOs can influence these aspects [49,50,51,52,53].

The concentration-dependent (1% and 5%) anti-Salmonella properties of the active EOs (Figure 1) were to be expected, but the unexpected result for patchouli oil, which was more effective at a lower concentration, might reflect a special mode of action for this EO. Alternatively, it might raise the possibility of different physicochemical properties during the experiments. One possible explanation is that lower concentrations of EOs could solubilise more effectively on the agar surface, spreading easily and evoking a more efficient antibacterial response [54]. We did not investigate this aspect but future studies might be dedicated to this issue. Despite this, we focused on finding essential oils with antimicrobial and antibiofilm properties that can cover different Salmonella isolates belonging to different genera.

To achieve the goals of the study, it was essential to create a reliable biofilm test. However, during the pilot studies it turned out that the combination of different factors had drastic effects on the biofilm formation of these 16 different isolates. It was not completely surprising, since the influencing effect on the biofilm formation of these serovariants on both biotic and abiotic surfaces was previously investigated using 96-well polystyrene plates, typically adhesive ones [15,55,56]. The increased biofilm formation capability of certain isolates (Figure 2, e.g., 1264, 1268, 1272, etc.) under various environmental conditions, using non-adhesive and suspension plates, highlights the fact that biofilm formation is not necessarily the most intensive when adhesive plates are used (Figure 2).

Nevertheless, the biofilm formation potential of the 16 distinct isolates belonging to the three *Salmonella serovars* on polystyrene surfaces was clearly demonstrated to depend not only on surface quality, but also on the isolate, temperature and medium (Figure 2). Some of our results resonate with previous research findings in which the serovar- and isolate-dependent biofilm formation of *Salmonellae* was described [57,58]. Cumulative data strongly suggest that biofilm formation is a complex process that has outcomes influenced by general aspects and individual features such as the presence and activity of regulators affecting stress responses, metabolic pathways, and the expression of matrix components and outer surface proteins. It may therefore be a more complex, multifactorial process than previously thought [59,60,61,62,63,64]. This was the reason we decided to analyze biofilm formation with different combinations in the first part of our study.

Temperature is a detrimental factor in the regulation of Salmonella, also affecting biofilm formation [65,66,67]. Our results are in accordance with previous studies, in that its formation is more pronounced at higher temperatures, such as 23 °C and 37 °C [67,68], but it can also be detected at 4 °C if a longer incubation time is applied [68,69,70]. The drastic increase in the extracellular matrix in the presence of MH-II, particularly in the case of *S.* Enteritidis and *S.* Infantis isolates at 4 °C (Figure 2), suggests the presence of key nutrients/factors in MH-II that might somehow activate cold stress more effectively, thereby inducing a more extensive biofilm formation [71,72]. While most biofilm assays are performed in commercial media such as LB, BHI and MH-II, deviations are more than likely to occur, which is why it might be difficult to compare the results of different studies.

The firm, relatively restrained, but balanced biofilm-supporting effect of chicken meat juice at 14 °C, 23 °C and 37 °C (Figure 2) makes it a fairly reliable biofilm supporter among the investigated Salmonella isolates and also in some recent studies [22,73,74]. Zhang et al.’s recent observation may explain this effect, since the supportive role of decanoic acid (a medium-chain fatty acid present in chicken meat) in this process was demonstrated [75]. The presence of decanoic acid (a 10-carbon fatty acid) was found to lead to increased cellular metabolism, acidification and ATP levels, resulting in increased biofilm formation [75]. Furthermore, the presence of residual organic debris, passed through during filtration, was also reported to facilitate an increased biofilm formation [22]. In a food industrial environment, the presence of organic matter is realistic and can contribute to adherence and biofilm formation on abiotic surfaces, such as stainless steel [76].

From practical point view, the combination of temperature, CMJ and different surfaces is the most important aspect to consider. This is consistent with the inhibitory effect on biofilms (Figure 5) when cells are exposed to the antibacterial agent during proliferation.

Comparing the effects of 0.1% EOs revealed that biofilms provide some protection against the antibacterial effects of several EOs (Figure 5 and Figure 6). This is represented by higher biofilm values after treatment, which are more pronounced in cases where MH-II was used as a carrier medium (Figure 6). The higher biofilm values in the case of MH-II-supported biofilms can be attributed to a number of factors. One possibility is that the composition and structure of biofilms are influenced by at least to two factors, such as the supportive effect of the medium in building a more densely structured biofilm with a different composition. On the other hand, hydrophobic molecules, such as lipids, can hinder the penetration of active components, while carbohydrates can bind to them. Future transcriptomic and proteomic analyses can reveal underlying mechanisms. However, dense extracellular DNA or proteins can absorb these antimicrobial compounds [77,78]. Despite the protective counter-effects in biofilms, cinnamon and clove were found to drastically disrupt the biofilm (Figure 6 and Figure 8) and are considered to be the most powerful EOs against *Salmonellae* that, in addition to their antibacterial and biofilm inhibitory effects, have the capacity to destroy mature biofilms.

Our study showed that certain essential oils, such as clove, oregano, thyme, and cinnamon, are still capable of exerting an active effect against microorganisms even after 24 h. Meanwhile, we also observed less stable essential oils whose efficacy had decreased somewhat compared to a 1 h treatment, such as peppermint and lemon balm. The third category includes essential oils whose effectiveness significantly declines over time, such as vanilla and vetiver (Figure 8).

Several factors may underlie these processes and may cause the concentration of the essential oil to fall into the subinhibitory range. One reason for this is that temperature promotes the volatilization of the active components, causing them to slowly leave the system. This no longer inhibits bacterial growth (as was typical of monoterpenes). Another cause could be oxidation, which may also be facilitated by temperature. Monoterpenes, such as thujone, are unstable and prone to both oxidation and volatility [49]. Mint is also prone to oxidation and evaporation. According to the literature, citral, geraniol, and citronellal are particularly unstable. With moderate volatility, 1,8-cineole, α-pinene, and tumerones are characterized by a gradual loss of efficacy among the phenylpropanoids. Sesquiterpenes, which are highly stable, and vanillin, have a more persistent effect. Resinous, heavy components can also be considered more stable [29,49,79,80].

Furthermore, two other options could also be considered. One is resistance. Considering the fact that EO treatment induces a stress condition, and that such conditions can have an influence on membrane protein and lipid composition—which influences fluidity and permeability, and thereby the permeability of EO compounds—this option cannot be excluded [81]. On the other hand, under unfavourable conditions, several bacteria, including Salmonellae, can switch to a slowed-down metabolic state and reach the viable but non culturable (VBNC) form. In this form, bacteria become protected, in a so-called zombie state, and in becoming relieved of harmful effects they become revitalized [82].

Recent studies demonstrated the potential of different treatments, such as pulsed light [83] and commercial disinfectants [21], but the effects of pulsed light can be masked by presenting organic matter, and chemical disinfectants can have disturbing flavours. EO-based products could be as effective since their antibacterial compounds are volatile [51] and therefore some have the ability to diffuse through the biofilm matrix [84], penetrate in deeper layers and evoke their antibacterial effects on the residing bacterial cells, thereby solving the severe challenges of food-processing in hospital facilities.

In our study, we highlighted that a few essential oils (EOs) can reliably kill Salmonella cells and destroy mature biofilms formed by these bacteria. However, we also revealed discrepancies relating to certain factors that strongly influence biofilm formation and the antibacterial effect of EOs in the case of MH-II. More thorough, molecular-based studies will reveal the underlying mechanisms and provide a more detailed understanding of the influential factors with practical relevance in the food industry.

We have to emphasize that although our results are encouraging, they are based on in vitro experiments. Future studies have to reveal contributing in vivo aspects, such as surface spreading, fatty environment, evaporation or the influence of the evaporation of EOs. Some recent studies have already addressed these issues in controlling foodborne pathogens [84,85]. Lower temperatures decreased the membrane fluidity of bacteria, meaning that the bacterial membrane became more rigid and therefore became more impermeable [29,86]. In the investigated temperature range this was not observed in case of Salmonellae, in contrast to *Listeria monocytogenes* [38]. The more rigid and consequently less permeable bacterial membrane might be somewhat compensated by the fact that EOs evaporate much less at lower temperatures; therefore, the effect of an EO lasts longer [80]. On the other hand, at low temperatures, oils become more viscous and their spread on a surface is less efficient and might penetrate into biofilms more slowly. Therefore, applications such as carrier solutions that can positively influence these effects would be ideal [87].

## 5. Conclusions

The aim of the study was to investigate the antibacterial and biofilm-inhibiting effects of 60 different essential oils (EOs) against Salmonella. The experiment began with the characterization of the general biofilm-forming properties of 16 isolates of the most common serotypes (*S.* Typhimurium, *S.* Infantis, and *S.* Enteritidis), followed by the testing of 60 different essential oils using the drop plate method. Subsequently, the experiments were continued with *S*. Typhimurium isolate 1268, with the aim of inhibiting both developing and mature biofilms.

The results show that biofilm formation is a complex process influenced by multiple factors, including temperature, nutrient composition, surface type, and the characteristics of the specific bacterial strain. More intense biofilm formation is observed at higher temperatures (23–37 °C), but it can also occur at lower temperatures with longer incubation times.

Ajowan, thyme, orange, clove, and oregano essential oils at a 0.1% concentration completely inhibited biofilm formation. More than half of the 60 essential oils tested reduced the optical density of mature biofilms to below 0.05 (OD: 0.15–0.36); ajowan, lime, palmarosa, thyme, oregano, and clove were the most effective, exhibiting antibacterial, biofilm-inhibiting, and biofilm-dispersing effects on all tested Salmonella isolates.

In the future, it would be advisable to identify the antibacterial components of the most effective essential oils and then investigate their mechanisms of action.

## Figures and Tables

**Table 1:** List of *Salmonella* serovariants, used in this study.

ID	Serovar	Origin	Sample	Date of the Isolation	Phage Type
**1250**	Enteritidis	chicken	feces	2010	PT4
**1251**	Enteritidis	chicken	feces	2010	PT8
**1261**	Enteritidis	chicken	feces	2010	PT1
**1263**	Enteritidis	chicken	feces	2010	PT21
**1264**	Enteritidis	chicken	feces	2010	n.a.
**1267**	Typhimurium	chicken	feces	2010	PT8
**1268**	Typhimurium	chicken	feces	2010	n.a.
**1272**	Typhimurium	chicken	feces	2010	PT99
**1293**	Typhimurium	chicken	feces	2010	PT104
**1294**	Typhimurium	chicken	feces	2010	PT2
**VB1/S1**	Infantis	chicken	appendix	2018	n.a.
**VB4/S1**	Infantis	chicken	appendix	2018	n.a.
**VB9/S1**	Infantis	chicken	appendix	2018	n.a.
**VB10/S1**	Infantis	chicken	appendix	2018	n.a.
**VB12/S1**	Infantis	chicken	appendix	2018	n.a.
**10383 (ATCC 14028)**	Typhimurium	chicken	liver and heart	1960	n.a.

## Data Availability

The original contributions presented in this study are included in the article/Supplementary Materials. Further inquiries can be directed to the corresponding author.

## Supplementary Materials

The following supporting information can be downloaded at: https://www.mdpi.com/article/10.3390/foods15091574/s1. Table S1: OD620 values of the biofilm inhibition experiments (0.1% EOs); Table S2: OD620 values of the biofilm inhibition experiments (0.1% EOs); Table S3: OD620 values of the biofilm degradation experiments (0.1% EOs); Figure S1: GC analyses of ajowan, clove, lime, palmarosa, oregano and thyme.
