# Supplementary material for: Factors Influencing Biofilm Formation of Salmonella spp. and the Biofilm-Degrading Potential of Essential Oils
Source: Foods. 2026 May 3;15(9):1574. doi: 10.3390/foods15091574 (PMC13164475; doi:10.3390/foods15091574)
Supplement: Supplementary file 1 [file foods-15-01574-s001.zip › Figure S1 GC analyses of ajowan, clove, lime, palmarosa, oregano and thyme.pdf]

# Compound comoposition evaluation of ajowain, clove, lime, palmarosa, oregano and thyme essential oils with SPME-GC-MS

## Sample: Ajowain

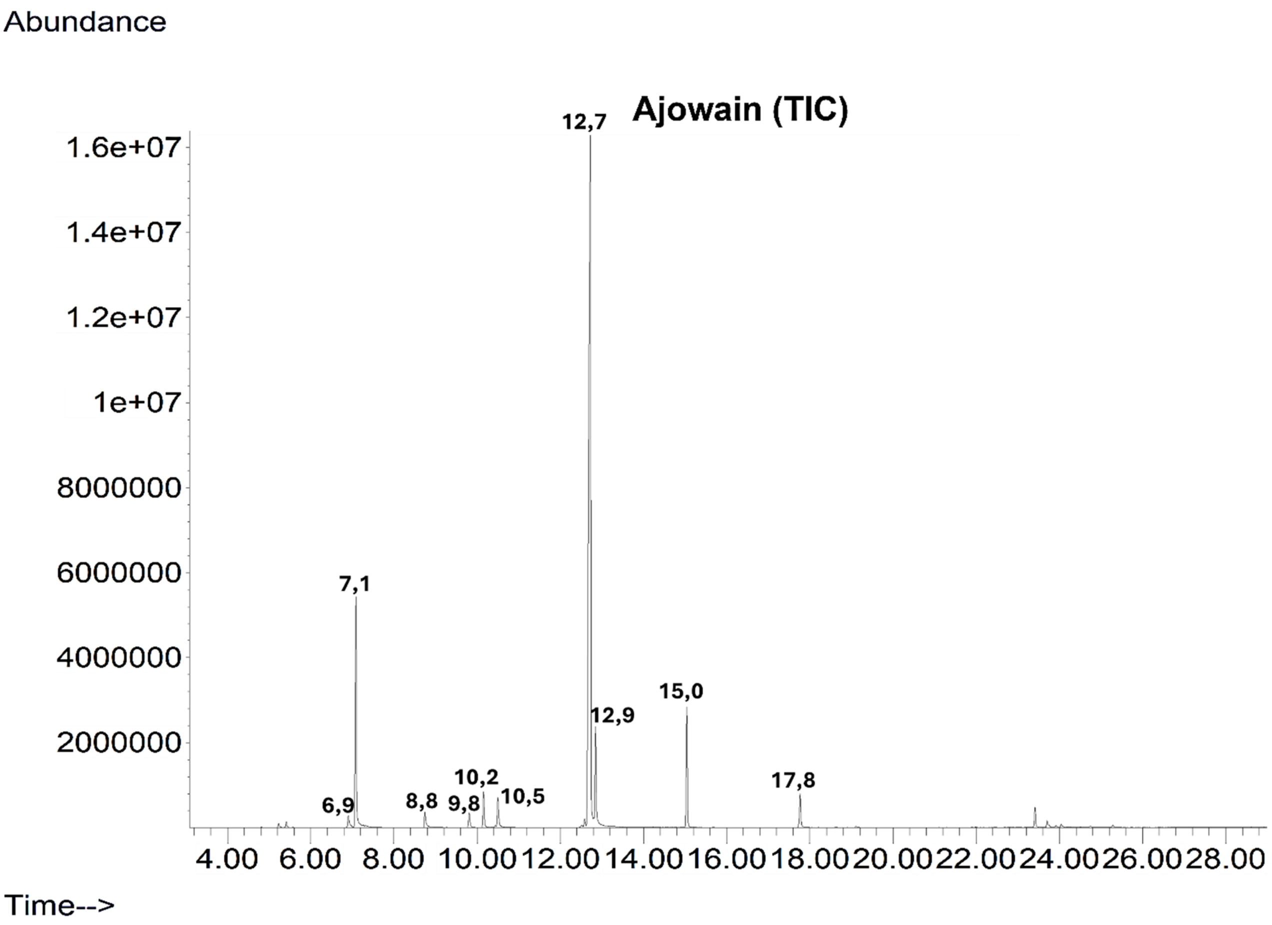


| Components | Retention time (min) | Relative abundance |
|---|---|---|
| α-Terpinene | 6.9 | 1.00 |
| *p*-Cymene | 7.1 | 12.31 |
| Linalool | 8.8 | 1.64 |
| Camphor | 9.8 | 1.67 |
| Borneol | 10.2 | 2.83 |
| Terpinen-4-ol | 10.5 | 2.71 |
| Thymol | 12.7 | 63.3 |
| Carvacrol | 12.9 | 6.16 |
| β-Caryophyllene | 15.0 | 6.64 |
| Caryophyllene oxide | 17.8 | 1.74 |

## Sample: Clove

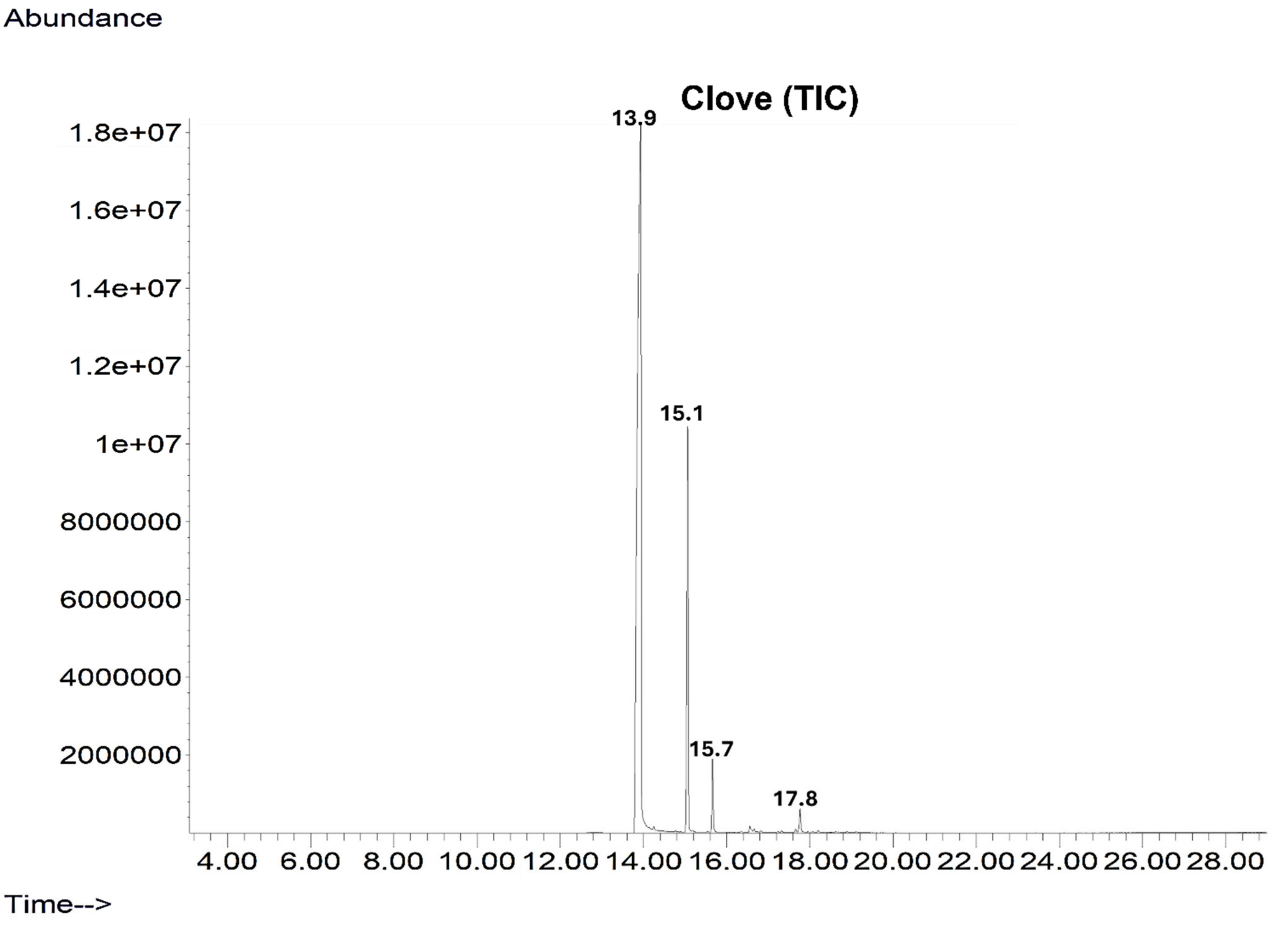


| Components | Retention time (min) | Area (%) |
|---|---|---|
| Eugenol | 13.9 | 79.52 |
| β-Caryophyllene | 15.1 | 16.73 |
| α-Humulene | 15.7 | 2.72 |
| Caryophyllene oxide | 17.8 | 1.03 |

**Sample: Clove RMO**

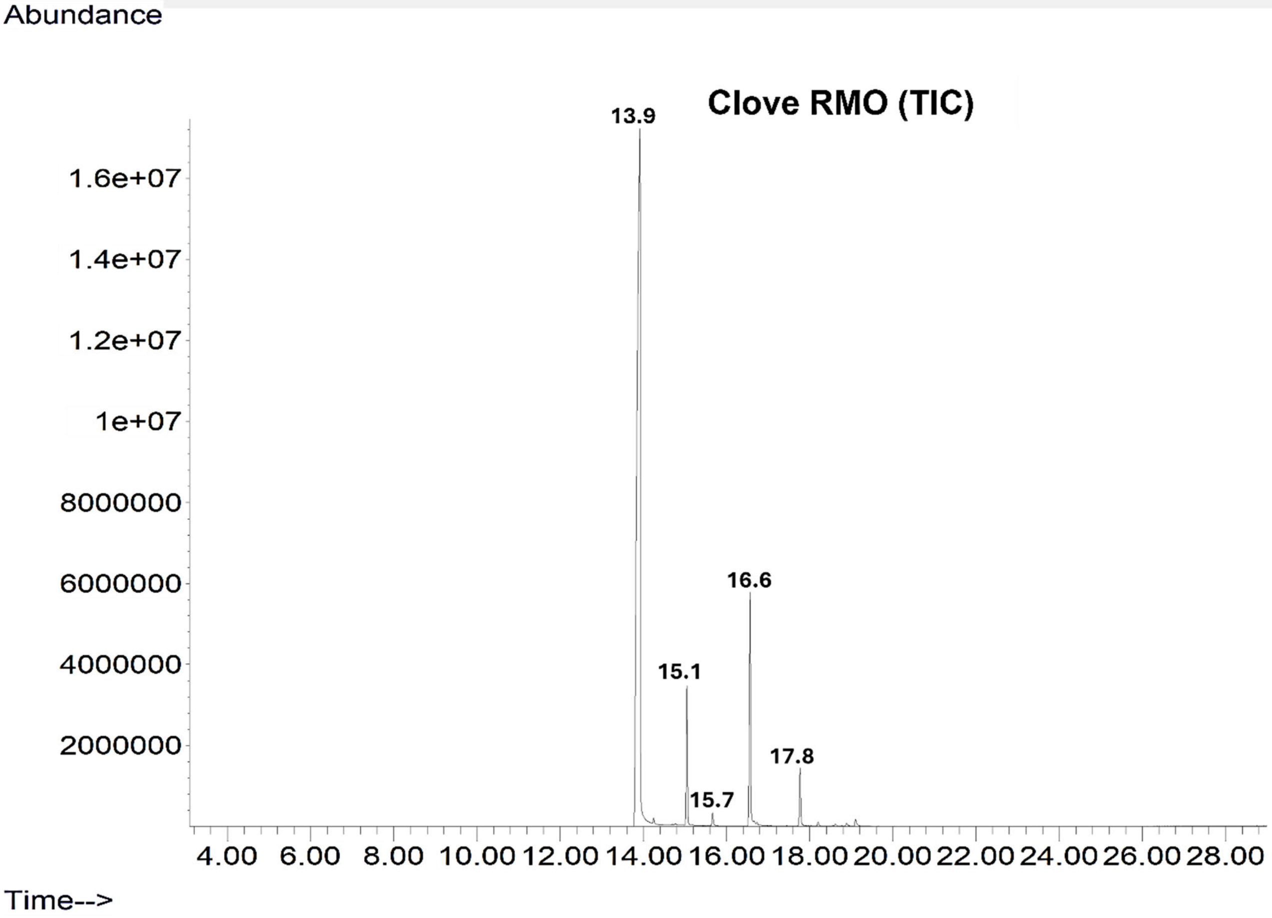


| Components | Retention time (min) | Area (%) |
|---|---|---|
| Eugenol | 13.9 | 80.28 |
| β-Caryophyllene | 15.1 | 5.81 |
| α-Humulene | 15.7 | 0.57 |
| Eugenyl acetate | 16.6 | 10.79 |
| Caryophyllene oxide | 17.8 | 2.56 |

**Sample: L.as per BP**

Abundance

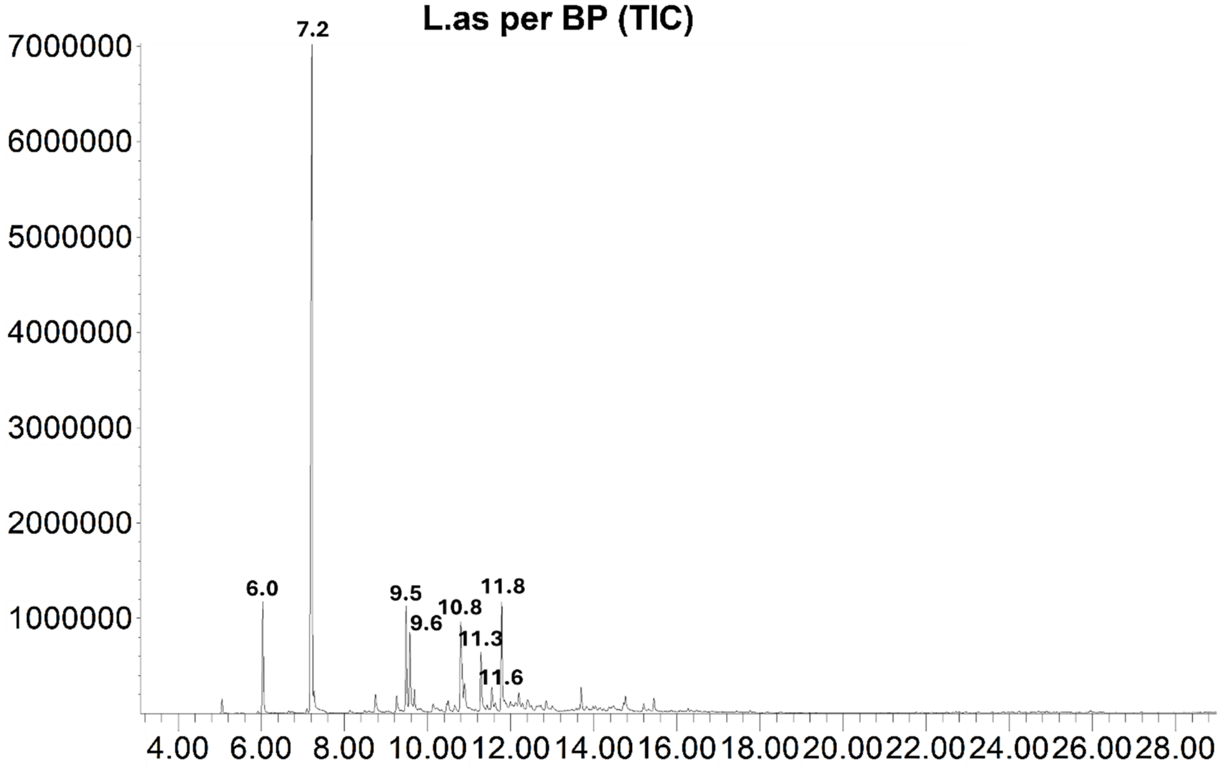


Time-->

| Components | Retention time (min) | Area (%) |
|---|---|---|
| β-Pinene | 6.0 | 8.16 |
| Limonene | 7.2 | 60.53 |
| Limonene oxide | 9.5 | 7.76 |
| Limonene epoxide | 9.6 | 5.83 |
| α-Terpineol | 10.8 | 10.41 |
| cis-Carveol | 11.3 | 5.15 |
| trans-Carveol | 11.6 | 1.73 |
| Carvone | 11.8 | 8.60 |

**Sample: Oregano**

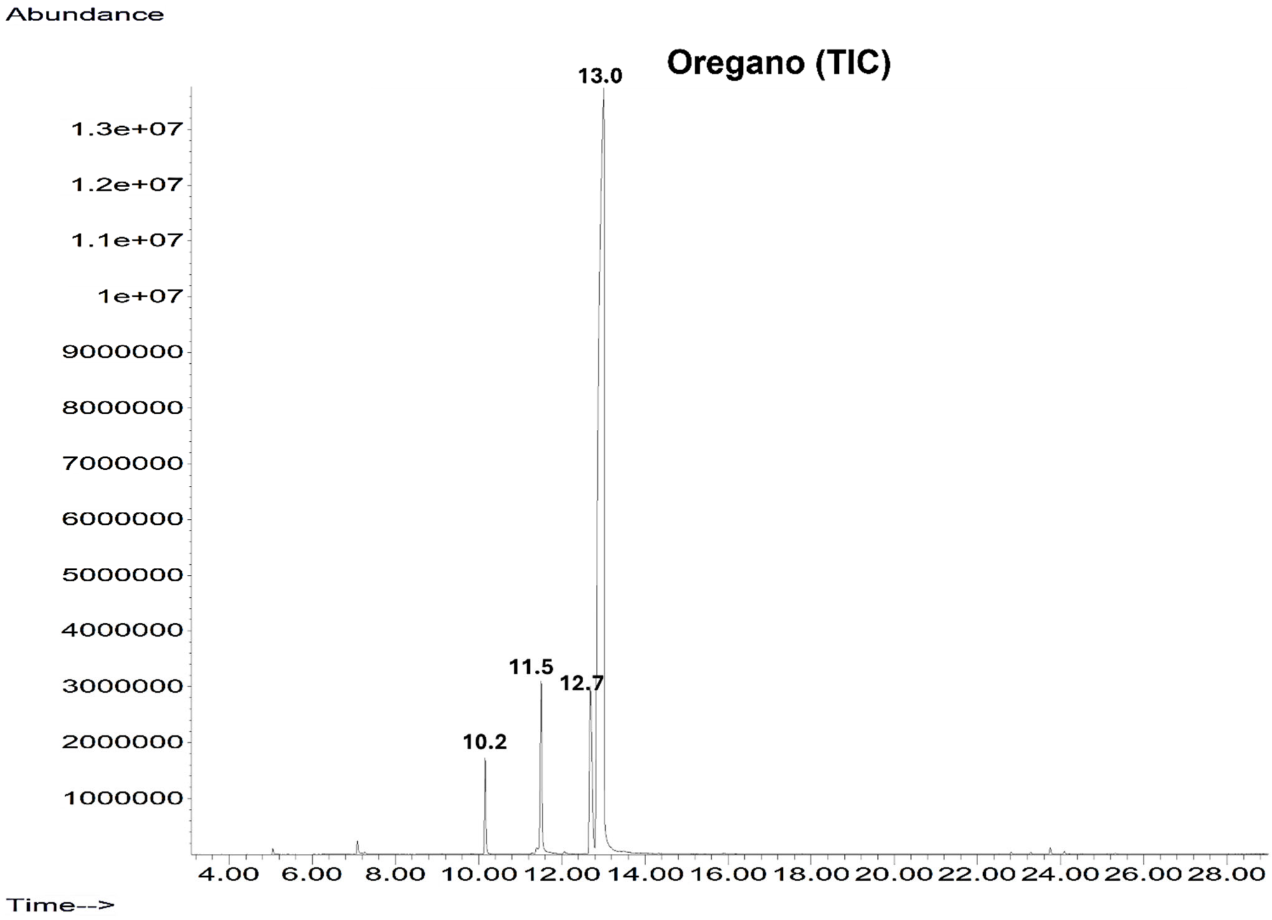


| Components | Retention time (min) | Area (%) |
|---|---|---|
| Borneol | 10.2 | 2.41 |
| β-Citronellol | 11.5 | 5.95 |
| Thymol | 12.7 | 8.33 |
| Carvacrol | 13.0 | 83.32 |

**Sample: Oregano RMO**

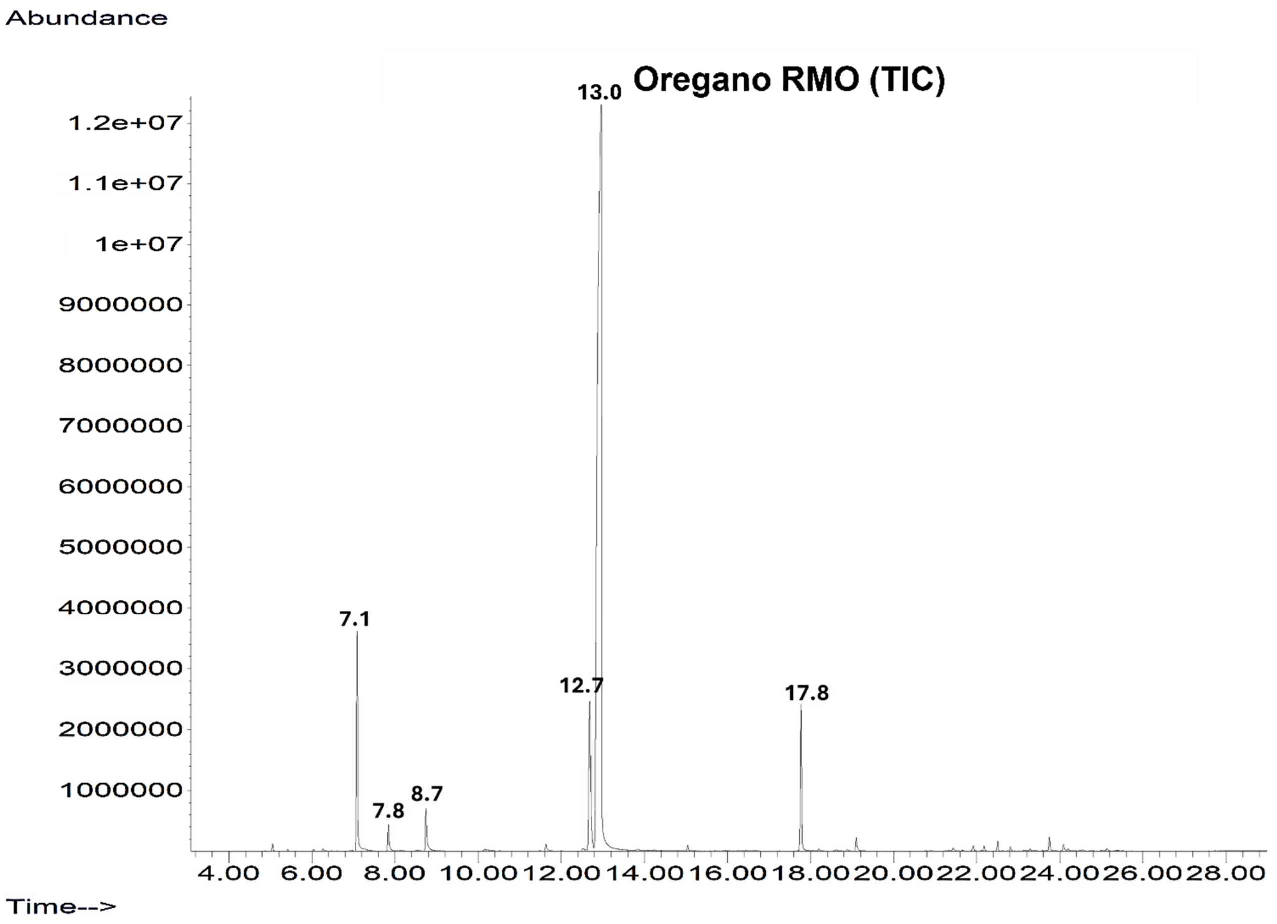


| Components | Retention time (min) | Area (%) |
|---|---|---|
| *p*-Cymene | 7.1 | 6.50 |
| γ-Terpinene | 7.8 | 0.85 |
| Linalool | 8.7 | 1.50 |
| Thymol | 12.7 | 7.19 |
| Carvacrol | 13.0 | 79.44 |
| Caryophyllene oxide | 17.8 | 4.53 |

**Sample: Palmarosa**

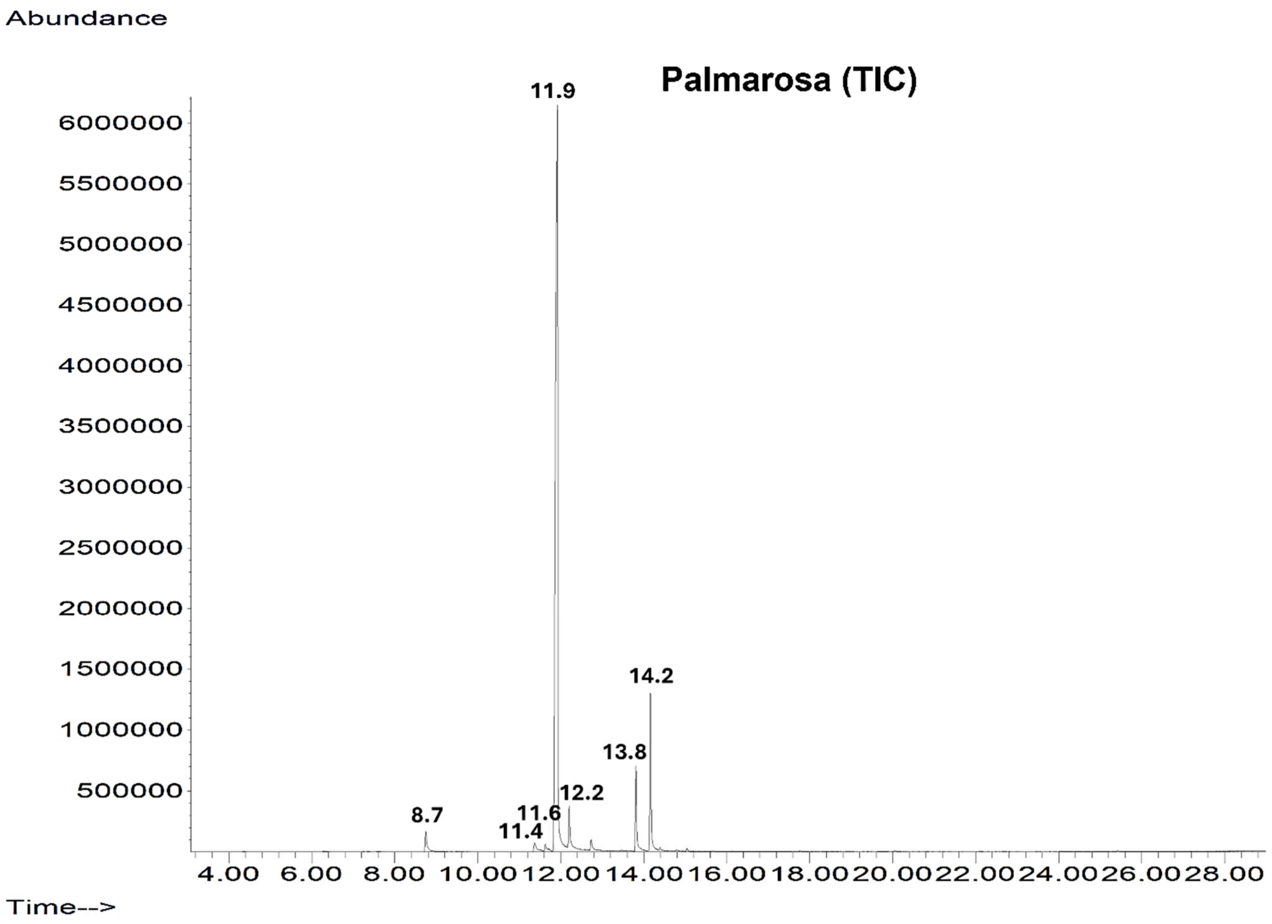


| Components | Retention time (min) | Area (%) |
|---|---|---|
| Linalool | 8.7 | 1.62 |
| Nerol | 11.4 | 1.05 |
| Neral | 11.6 | 0.51 |
| Geraniol | 11.9 | 79.79 |
| Geranial | 12.2 | 3.28 |
| Neryl acetate | 13.8 | 5.04 |
| Geranyl acetate | 14.2 | 8.69 |

**Sample: Thyme**

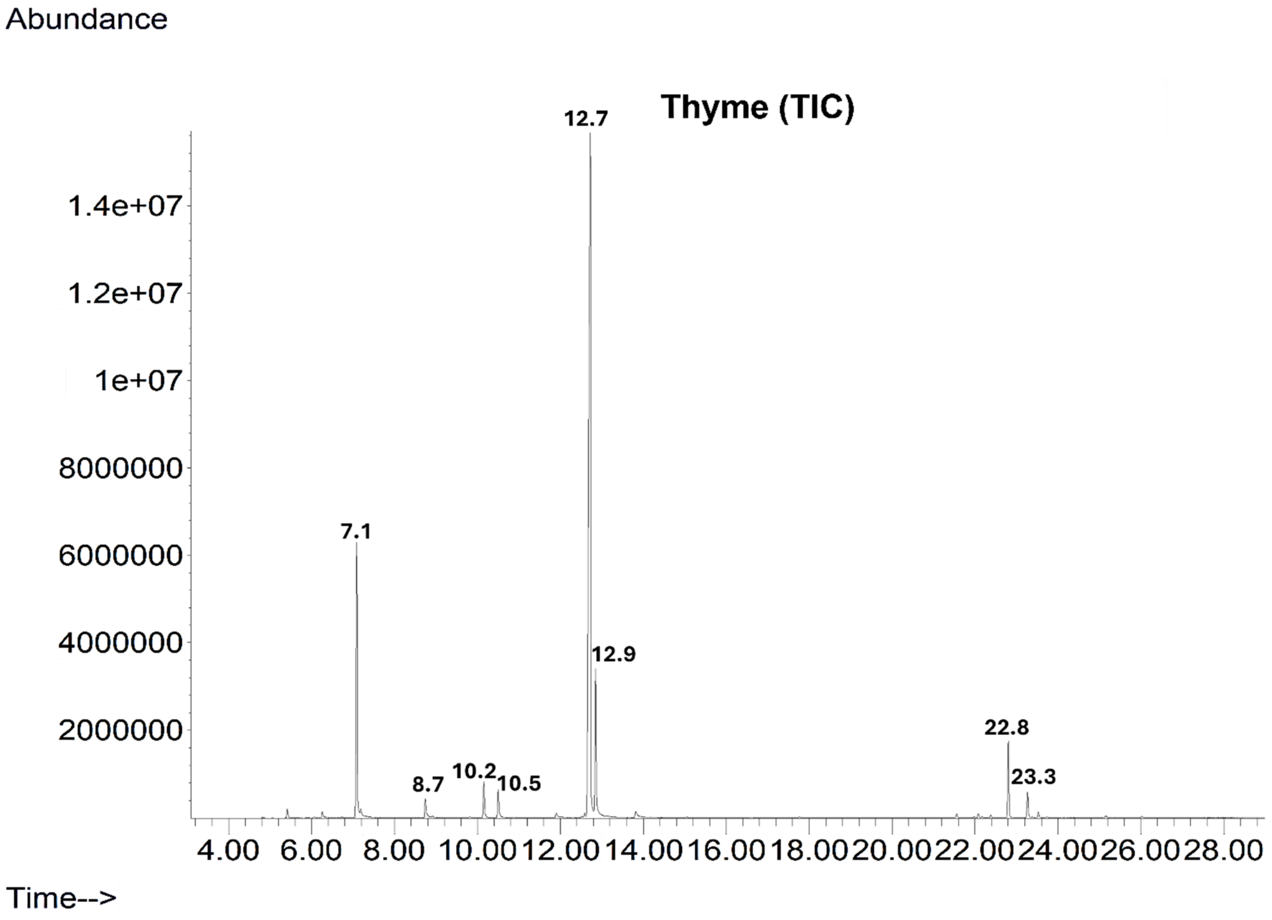


| Components | Retention time (min) | Area (%) |
|---|---|---|
| *p*-Cymene | 7.1 | 15.13 |
| Linalool | 8.7 | 1.35 |
| Borneol | 10.2 | 2.15 |
| Terpinen-4-ol | 10.5 | 1.91 |
| Thymol | 12.7 | 64.76 |
| Carvacrol | 12.9 | 8.89 |
| unknown | 22.8 | 4.31 |
| unknown | 23.3 | 1.53 |

**Supplementary Figure S4. SPME-GC-MS analysis of the ajowain, clove, lime, palmarosa, oregano and thyme essential oils**

1 µL of essential oil was placed into a 20 mL headspace (HS) vial sealed with a silicone/PTFE septum. Static headspace solid-phase microextraction (sHS-SPME) was performed using a CTC Combi PAL autosampler (CTC Analytics AG, Zwingen, Switzerland). After a 5-minute incubation at 100 °C, a 65 µm StableFlex divinylbenzene/carboxen/polydimethylsiloxane (DVB/CAR/PDMS) SPME fiber (Supelco, Bellefonte, PA, USA) was exposed to the headspace of the sample. Extraction was carried out for 20 minutes at 100 °C, followed by thermal desorption in the injector of the gas chromatograph at 250 °C for 1 minute. Injection was performed in split mode

with a split ratio of 1:90. The fiber was subsequently cleaned and conditioned in high-purity nitrogen at 250 °C for 15 minutes.

The analysis was carried out on an Agilent 6890N/5973N GC-MSD system (Santa Clara, CA, USA) equipped with a Supelco SLB-5MS capillary column (30 m × 250 μm × 0.25 μm). After an initial 3-minute isothermal period, the column temperature was programmed from 60 to 250 °C at a rate of 8 °C/min, and the final temperature was held for 1 minute. The carrier gas was high-purity helium (6.0), with a flow rate of 1.0 mL/min (37 cm/s) in constant-flow mode. Detection was performed using a quadrupole mass-selective detector in electron-ionization mode (70 eV), in full-scan mode (41–500 amu, 3.2 scans/s). Data were processed using MSD ChemStation D.02.00.275 software (Agilent). For quantitative identification, retention times and mass spectra were compared with standards and the NIST 2.0 library, while percentage evaluation was performed using area normalization.
