# Supplementary material for: Factors Influencing Biofilm Formation of Salmonella spp. and the Biofilm-Degrading Potential of Essential Oils
Source: Foods. 2026 May 3;15(9):1574. doi: 10.3390/foods15091574 (PMC13164475; doi:10.3390/foods15091574)
Supplement: Supplementary file 1 [file foods-15-01574-s001.zip › Table S1 OD620 values of the biofilm inhibition experiments (0.1% EOs).pdf]

List of the essential oils, used in this study. Abbreviatins: A.G. Ind.: AG Industries, Noida-UP, India; RMO: Rocky Mountain Oils, Orem-Utah, USA

| Name of the essential oil | Binomial nomenclature | Industries |
|---|---|---|
| Ajowain Oil | *Trachyspermum ammi* | A.G. Ind. |
| Anethole oil | *Foencukum vulgare mill* | A.G. Ind. |
| Basil oil | *Ocimum basalicum* | A.G. Ind. |
| Bay oil | *Laurus nobilis* | A.G. Ind. |
| Black pepper oil | *Piper nigrum* | A.G. Ind. |
| Calamus oil | *Acorus calamus* | A.G. Ind. |
| Chamomilla | *Matricaria Chamomilla* | A.G. Ind. |
| Cajeput oil | *Melaleuca leucadendron* | A.G. Ind. |
| Cedarwood oil | *Juniperus ashei* | A.G. Ind. |
| Cardamom oil | *Elettaria cardamomum* | A.G. Ind. |
| Cinnamon oil | *Cinnamomum zeylanicum* | A.G. Ind. |
| Cinnamon leaf oil | *Cinnamomum zeylanicum* | A.G. Ind. |
| Citronella oil | *Cymbopogon nardus* | A.G. Ind. |
| Clary Sage oil | *Salvia sclarea* | A.G. Ind. |
| Clove | *Syzygium aromaticum* | RMO |
| Cypress oil | *Cupressus sempervirens* | A.G. Ind. |
| Dill seed oil | *Anethum graveolens* | A.G. Ind. |
| Eucalyptus oil | *Eucalyptus globulus* | A.G. Ind. |
| Fennel oil | *Foeniculum vulgare* | A.G. Ind. |
| Fenugreek oil | *Trigonella foenum-graecum* | A.G. Ind. |
| Frankincense oil | *Boswellia serrata* | A.G. Ind. |
| Geranium oil | *Pelargonium graveolens* | A.G. Ind. |
| Ginger oil | *Apium graveolens* | A.G. Ind. |
| Grapefruit oil | *Citrus Paradisi* | A.G. Ind. |
| Jasmine oil | *Jasminum gradiflora* | A.G. Ind. |
| Juniper oil | *Juniperus communis* | A.G. Ind. |
| Lavender oil Bulgarian | *Lavandula angustifolia* | A.G. Ind. |
| Lemon oil | *Citrus limanum* | A.G. Ind. |
| Lemon Eucalyptus oil | Eucalyptus citriodora | A.G. Ind. |
| Lavender oil Himalyan | *Lavandula angustifolia* | A.G. Ind. |
| Lime oil | *Citrus aurantifolia* | A.G. Ind. |
| Lime oil as per BP | *Citrus aurantifolia* | A.G. Ind. |
| Melissa oil | *Melissa officinalis* | A.G. Ind. |
| Minth oil | *Mentha spicata* | A.G. Ind. |
| Myrtle oil | *Myrtus communis* | A.G. Ind. |
| Neroli oil | *Citrus aurantium* | A.G. Ind. |
| Nutmeg oil | *Myrstica fragans* | A.G. Ind. |
| Orange | *Citrus sinensis* | A.G. Ind. |
| Oregano | *Origanum vulgare* | RMO |
| Palma Rosa oil | *Cymbopogon martinii* | A.G. Ind. |

| | | |
|---|---|---|
| Patchouli oil | *Pogostemon cablin* | A.G. Ind. |
| Pepperminth oil | *Mentha piperita* | A.G. Ind. |
| Pettitgrain oil | *Citrus aurantium* | A.G. Ind. |
| Pine oil | *Pinus sylvestris* | A.G. Ind. |
| Ravensara oil | Ravensara aromatica | A.G. Ind. |
| Rose Geranium oil | *Pelargonium graveolens* | A.G. Ind. |
| Saffron oil | *Crocus sativus* | A.G. Ind. |
| Sandalwood oil | *Santalum album* | A.G. Ind. |
| Sage oil | *Salvia officinalis* | A.G. Ind. |
| Tarragon oil | *Artemisia dracunculus* | A.G. Ind. |
| Tea Tree oil | *Melaleuca alternifolia* | A.G. Ind. |
| Thuja oil | *Thuja occidentalis L.* | A.G. Ind. |
| Tolu Balsam | *Myroxylon balsamum* | A.G. Ind. |
| Turmeric oil | *Curcuma longa* | A.G. Ind. |
| Wintergreen oil | *Gaultheria fragrantissima* | A.G. Ind. |
| Ylang Ylang oil | *Cananga odorata var genuina* | A.G. Ind. |
| Vanilla | *Vanilla planifolia* | A.G. Ind. |
| Vetiver oil | *Vetiveria zizanoides* | A.G. Ind. |
| Mace oil | *Myristica fragrans* | A.G. Ind. |
| Thyme | *Thymus vulgaris* | A.G. Ind. |
